# Noninvasive Focal Gene Delivery into the Cerebellum of Non‐Human Primates using Focused Ultrasound

**DOI:** 10.1002/advs.75307

**Published:** 2026-04-20

**Authors:** Noelia Esteban‐García, José A. Pineda‐Pardo, Inés Trigo‐Damas, Marta Gutiérrez, Marta Castillo‐Ortiz, Megan Carrillo, Alejandro Reinares‐Sebastian, Víctor Medina‐Chavarrías, Itay Rachmilevitch, Masahiko Takada, José A. Obeso, Javier Blesa

**Affiliations:** ^1^ HM CINAC (Centro Integral de Neurociencias Abarca Campal) Hospital Universitario HM Puerta del Sur, HM Hospitales Madrid Spain; ^2^ Instituto de Investigación Sanitaria HM Hospitales Madrid Spain; ^3^ Network Center for Biomedical Research on Neurodegenerative Diseases (CIBERNED) Instituto Carlos III Madrid Spain; ^4^ PhD Program in Neuroscience, Universidad Autónoma de Madrid‐Cajal Institute Madrid 28029 Spain; ^5^ Centro Universitario HM Hospitales de Ciencias de la Salud (CUHMED) Universidad Camilo José Cela Madrid Spain; ^6^ PhD Program in Technologies for Health and Well‐being Polytechnic University of Valencia Valencia Spain; ^7^ Molecular Imaging Technologies Research Institute (I3M) Polytechnic University of Valencia Valencia Spain; ^8^ Insightec Ltd. Haifa Israel; ^9^ Department of Neurology Graduate School of Medicine Osaka University Suita Osaka Japan

**Keywords:** blood–brain barrier, cerebellum, focused ultrasound, gene therapy, non‐human primates

## Abstract

Cerebellar degenerations are a heterogeneous group of disorders that pose significant clinical challenges, and no effective therapies are currently available to halt or slow their progression. Gene therapy offers important therapeutic potential for brain disorders; however, its clinical translation is hindered by critical obstacles, particularly the blood–brain barrier, which limits systemic delivery of therapeutic genes to the brain, an issue especially pronounced in primates. Here, we evaluated the use of low‐intensity focused ultrasound combined with intravenously administered microbubbles to transiently open the blood–brain barrier in the cerebellum of macaque monkeys, thus enabling targeted delivery of adeno‐associated virus‐based gene therapy vectors. Two vector types (scAAV9‐CBA‐GFP and ssAAV9‐CMV‐mCherry) were administered systemically, and transgene expression was analyzed to assess delivery efficiency and cell‐type distribution. We achieved successful, non‐invasive delivery of both vector types to the cerebellum with high spatial precision. Administration of ssAAV9‐CMV‐mCherry vector resulted in robust transduction of virtually all neurons within the targeted deep cerebellar nuclei. These findings provide a promising and translationally relevant strategy for developing gene‐delivery approaches for cerebellar and other neurodegenerative disorders and represent a step forward in advancing the use of focused ultrasound to achieve efficient and less invasive gene delivery to the brain.

## Graphical abstract

Focal and non‐invasive viral vector delivery in non‐human primates remains a major challenge in translational neuroscience. Low‐intensity focused ultrasound was used to transiently open the blood–brain barrier and enable targeted gene delivery to the cerebellum. Using two distinct viral vectors, widespread transduction was achieved throughout the ultrasound‐targeted hemisphere. These findings demonstrate a highly efficient and spatially precise strategy for non‐invasive gene delivery in primates.

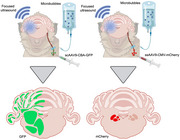

## 1 Introduction

Neurodegenerative disorders are diseases that typically exhibit relatively specific initial cell loss, with progression toward more widespread pathology. These diseases not only cause progressive disability but also impose a substantial socioeconomic burden. Several newer therapeutic options are under development, but their efficacy is often limited by poor penetration through the blood–brain barrier (BBB) [1]. Meanwhile, gene therapy holds considerable promise as a potential treatment for neurodegenerative disorders. However, traditional gene delivery approaches typically involve invasive surgical procedures, such as craniotomy and direct intraparenchymal injection, which are further complicated by the need to reach multiple or anatomically complex brain regions [2]. Especially in human patients, targeting deep or poorly accessible structures poses major technical and safety challenges.

Low‐intensity focused ultrasound combined with intravenously administered microbubbles (LIFU‐MB) offers a safe and noninvasive method of BBB opening to enhance drug delivery [3, 4, 5, 6]. We have recently demonstrated that LIFU‐MB can safely and transiently disrupt the BBB in both non‐human primates and humans [7, 8, 9, 10, 11]. In adult macaque monkeys, this technique enabled targeted gene transduction in subcortical regions implicated in Parkinson's disease (PD) by using adeno‐associated virus (AAV) vectors serotype 9‐based vectors, i.e., AAV9‐PHP.eB and AAV9.2‐PHB.eB vectors [7].

Building on these findings, we now show that LIFU‐MB can also facilitate precise, non‐invasive gene delivery of both self‐complementary and single‐stranded AAV9 (scAAV9 and ssAAV9) vectors into the cerebellum, specifically targeting the deep cerebellar nuclei in adult macaques. This is particularly relevant given that many cerebellar disorders, such as Friedreich's ataxia, have a genetic basis and may benefit from gene therapy approaches via viral vectors [12].

## 2 Results

### 2.1 Blood–Brain Barrier Openings in the Cerebellum

Transcranial LIFU‐MB under magnetic resonance imaging (MRI) guidance was applied to four monkeys (M1 to M4). The targeted region included the left cerebellar hemisphere, with the right hemisphere served as a control. For these interventions, we used a previously described protocol that demonstrated the feasibility of BBB opening in PD patients [8, 9, 11]. All procedures were successfully completed in the four macaques. Table 1 summarizes all given sonications, including acoustic power, duration, grid spacing, and size (number of spots), and the programmed and achieved target cavitation dose. Delivered acoustic power values ranged from 1.7 to 5.2 W across the group. While monkeys M2–M4 needed average powers between 2.4 and 3.2 W, M1 required a slightly higher value (4.6 W) to reach the programmed target cavitation dose. Each experiment included 7 targets, except for M1, which had 5 targets, with 10–31 grid spots per target, leading to consistent coverage of the left cerebellar hemisphere. The programmed target cavitation dose averaged 0.30 in M1 and M2, 0.18 in M3, and 0.13 in M4. The achieved cavitation dose closely matched these values, 0.27, 0.28, 0.17, and 0.11, respectively, with an average deviation ≤0.03, confirming the accurate and stable dose delivery by the closed‐loop controller (Table S1).

**TABLE 1:** Summary of sonication parameters for the four macaque experiments. The table includes the effective average acoustic power, average sonication duration, number of targets, average number of grid spots per target, the console‐defined target cavitation dose, and the achieved cavitation dose. Values are reported as mean (standard deviation) and [min–max]. M1* refers to a preliminary calibration experiment performed on macaque 1, which was conducted solely to tune acoustic power to cavitation dose by sonicating cortical regions with no relevance to the outcomes of the present study; therefore, M1* is not part of this experimental series.

	Power (W)	Duration (s)	Number of targets	Spots per‐target	Target Cavitation Dose	Cavitation Dose
M1*	1.9 (0.9) [0.3–3]	10	12	8.8 (1.1) [4–9]		0.07 (0.09) [0.01–0.38]
M1	4.6 (0.9) [3.2–5.2]	100	5	24.5 (3.4) [13–21]	0.30	0.27 (0.01) [0.27–0.28]
M2	3.2 (0.8) [2.1–4.4]	100	7	23.9 (7.8) [10–31]	0.30	0.28 (0.02) [0.24–0.29]
M3	2.4 (0.6) [1.7–3.2]	100	7	22.4 (2.9) [13–19, 27, 28]	0.18 (0.06) [0.15–0.3]	0.17 (0.06) [0.13–0.29]
M4	3.1 (0.7) [1.9–3.9]	100	7	24.1 (2.4) [13–20]	0.13 (0.03) [0.10–0.15]	0.11 (0.03) [0.08–0.14]

**FIGURE 1:**
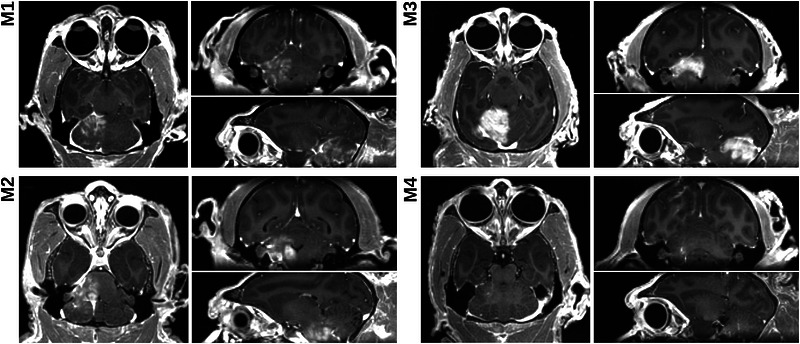
Contrast‐enhanced MRI scans following blood–brain barrier (BBB) opening. For each animal (M1‐M4), axial (left), coronal (top right), and sagittal (bottom right) T1‐weighted images are shown. Regions of BBB opening correspond to areas of increased signal enhancement, indicating contrast agent uptake.

**FIGURE 2:**
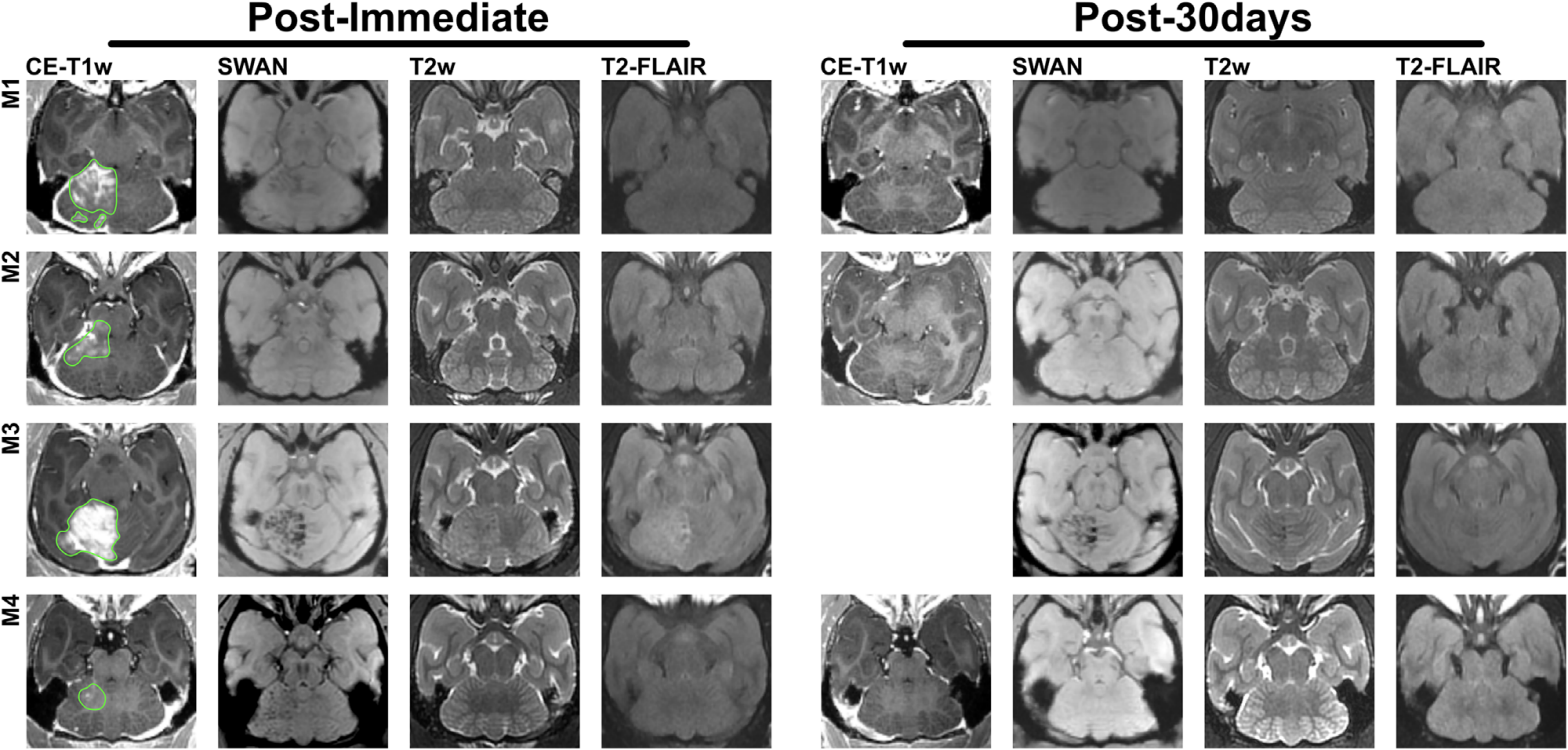
Immediate and 30‐day follow‐up assessment of blood–brain barrier (BBB) opening in the cerebellum. MRI sequences included contrast‐enhanced T1‐weighted imaging (CE‐T1w) with gadolinium (Gd), susceptibility‐weighted imaging (SWAN), T2*‐weighted imaging (T2‐w), and fluid‐attenuated inversion recovery (T2‐FLAIR). Images are shown in axial view at the slice corresponding to the maximal extent of BBB disruption, as indicated by Gd extravasation for all subjects (M1–M4). CE‐T1w for M3 is not available. Imaging was performed immediately after treatment (Post‐immediate) and at 30‐day follow‐up (Post‐30 days).

We assessed BBB opening success by inspection of T1‐weighted images after administration of gadolinium (Gd), a compound that does not otherwise cross the BBB (Figure 1). BBB openings were consistently obtained; however, post‐contrast enhancement was clearly heterogeneous, with some regions showing markedly stronger signal than others. In some cases (such as M1), enhancement appeared more intense within the natural cerebellar fissures, where cerebrospinal fluid‐filled spaces interdigitate with the parenchyma. This pattern suggests preferential accumulation or redistribution of contrast agent within these anatomical recesses. Manual segmentation of contrast enhancement revealed BBB openings of 1.90 cm^3^ for M1, 1.04 cm^3^ for M2, 3.37 cm^3^ for M3, and 0.08 cm^3^ for M4 (Figure 1). In the latter case, the markedly low Gd enhancement may reflect a procedural issue during Gd injection, which cannot be ruled out. No BBB disruption was observed elsewhere. The experimental procedure was safe and well tolerated by all animals except for one monkey (M3), the one with the largest BBB opening, who developed transient contralateral hemiparesis that was treated with methylprednisolone for 4 days until complete recovery. M3 showed detectable signal changes in the immediate post‐treatment scans, with focal edema at the sonication site on both T2‐weighted fast spin‐echo (T2w) and fluid‐attenuated inversion recovery (T2‐FLAIR) images, which markedly decreased by the 30‐day follow‐up. Susceptibility‐weighted angiography (SWAN) images revealed hypointensities in all monkeys immediately after BBB opening; these largely diminished in M1, M2, and M4, whereas in M3 they remained clearly visible at 30 days (Figure 2).

### 2.2 Delivery of Viral Vectors

To examine the potential therapeutic value of LIFU‐MB induced BBB opening for gene delivery into the cerebellum, two monkeys (M1 and M2) underwent intravenous administration of the self‐complementary AAV9 (scAAV9) vector that expresses green fluorescent protein (GFP) under the chicken β‐actin promoter (scAAV9‐CBA‐GFP), while the other two monkeys (M3 and M4) received administration of the single strand AAV9 (ssAAV9) vector that expresses *mCherry* under the cytomegalovirus promoter (ssAAV9‐CMV‐mCherry). The absence of neutralizing antibodies against AAV9 was confirmed in all animals before viral vector administration. Four weeks after administration, the monkeys were euthanized, and the brains were analyzed postmortem to evaluate GFP or *mCherry* expression, along with Nissl and other marker staining.

Both hemispheres (opened and control) appeared visually intact upon macroscopic examination. Nissl staining revealed occasional neuronophagy and altered vascular morphology in M1, M2, and M4 (Figure S1). In the case of M3, in addition to these findings, focal coagulative necrosis was observed surrounding the cortex and deep cerebellar nuclei (Figure S1E). Additional staining with the microglial marker ionized calcium‐binding adapter molecule 1 (Iba1) confirmed focal microgliosis predominantly surrounding blood vessels, although Iba1+ cells generally exhibited normal morphology within the targeted areas (Figure S2). Glial Fibrillary Acidic Protein (GFAP) staining showed no evident changes in M1, M2, and M4; only M3 displayed a slight increase in astroglial activation (Figure S3).

**FIGURE 3:**
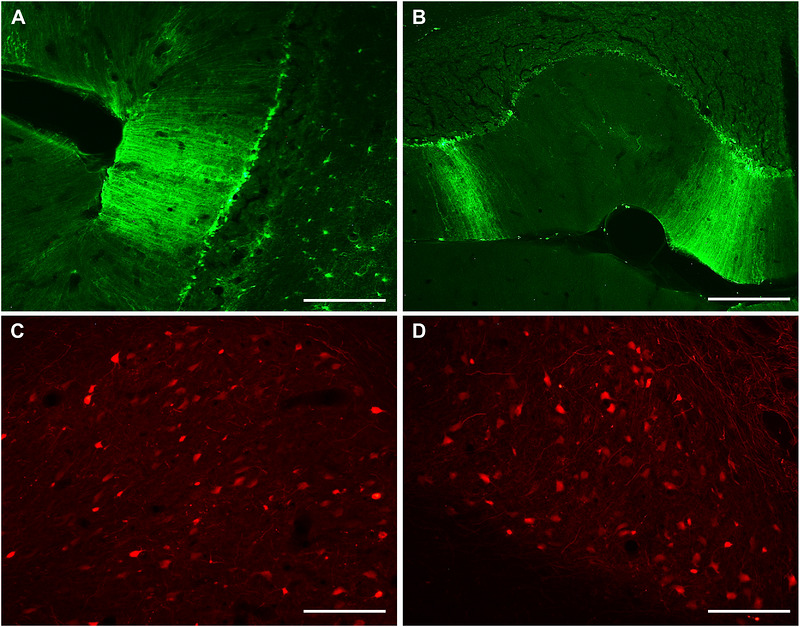
Native fluorescence of scAAV9‐CBA‐GFP and ssAAV9‐CMV‐mCherry. High‐magnification coronal sections within the BBB‐opened hemispheres show native fluorescence in the cerebellar cortex of (A) M1 and (B) M2 after injection with scAAV9‐CBA‐GFP, and in the deep cerebellar nuclei of (C) M3 and (D) M4 after injection with ssAAV9‐CMV‐mCherry. Scale bar: 200 µm.

Histological analysis of both GFP and *mCherry* demonstrated successful delivery of both vectors, confined to the target area. Expression of GFP and *mCherry* was confirmed both by native fluorescence (Figure 3) and by immunohistochemistry and immunofluorescence histochemistry using two different antibodies for each marker (for details, see below).

**FIGURE 4:**
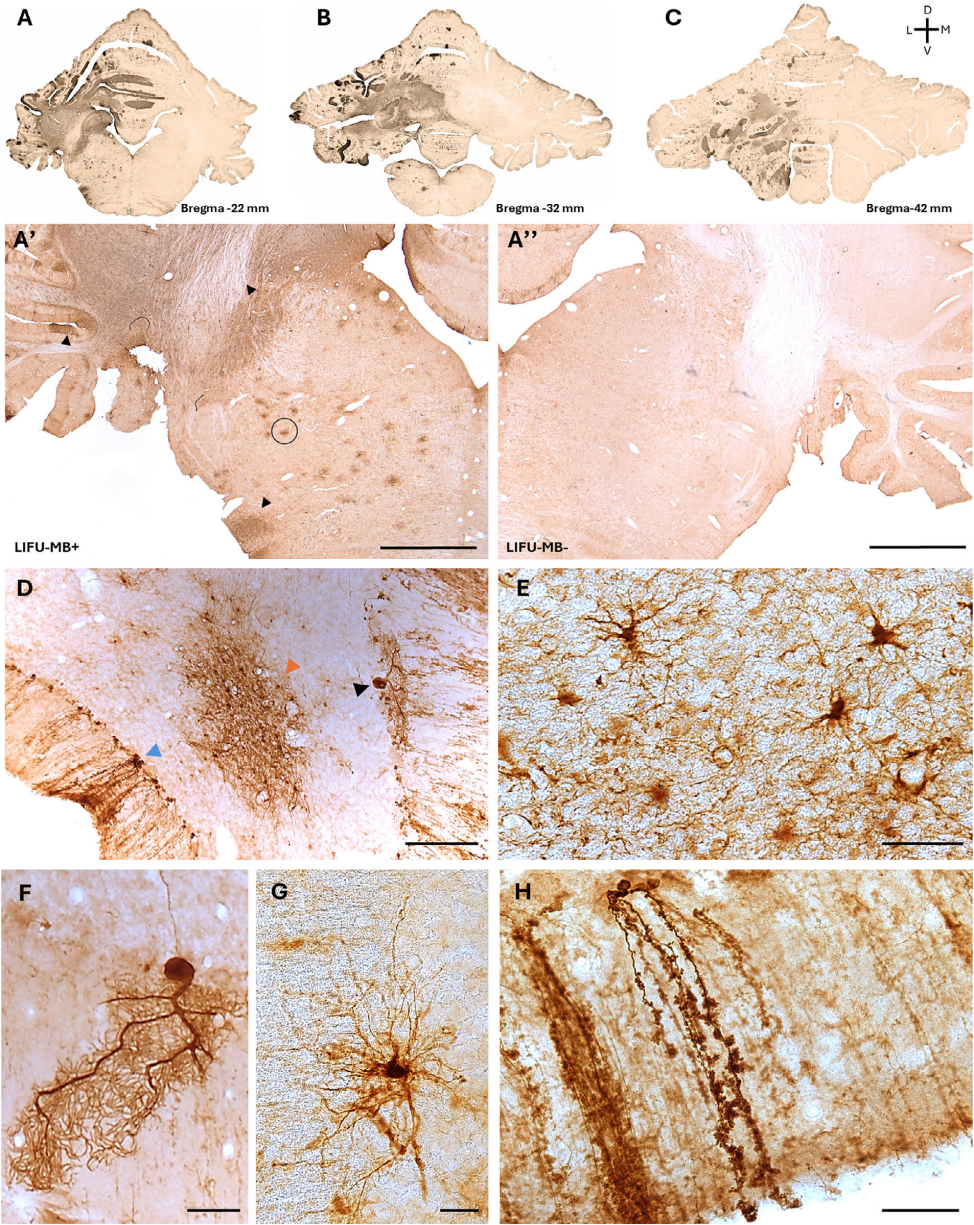
Cerebellar delivery of scAAV9‐CBA‐GFP in M1. (A–C) Pseudocolor, low‐magnification coronal sections through the rostrocaudal axis illustrate the distribution and relative density of GFP immunoreactivity. The bregma level of each section, based on the stereotaxic atlas of Paxinos et al. [13] is indicated in the lower right corner of each panel. Mid‐magnification views of the BBB‐opened cerebellar hemisphere (A′) and the contralateral hemisphere (A″) highlight widespread GFP labeling in the injected side, contrasting with limited expression in the contralateral hemisphere. Abundant patches of GFP‐immunoreactive fibers and labeled cell bodies are visible (arrows and circles). (D) Representative images show GFP immunostaining in Purkinje cells (black arrows), Bergmann glia (blue arrows), and other glial cells (orange arrows). (E) High‐magnification images show GFP expression in astrocytes within the white matter, (F) Purkinje cells in the Purkinje layer, (G) astrocytes in the granular layer with numerous fine, highly branched processes, (H) and Bergmann glia in the molecular layer. D: dorsal; V: ventral; M: medial; L: lateral. Scale bars: 2 mm (A′, A″), (D) 200 µm, (E–H) 50 µm.

**FIGURE 5:**
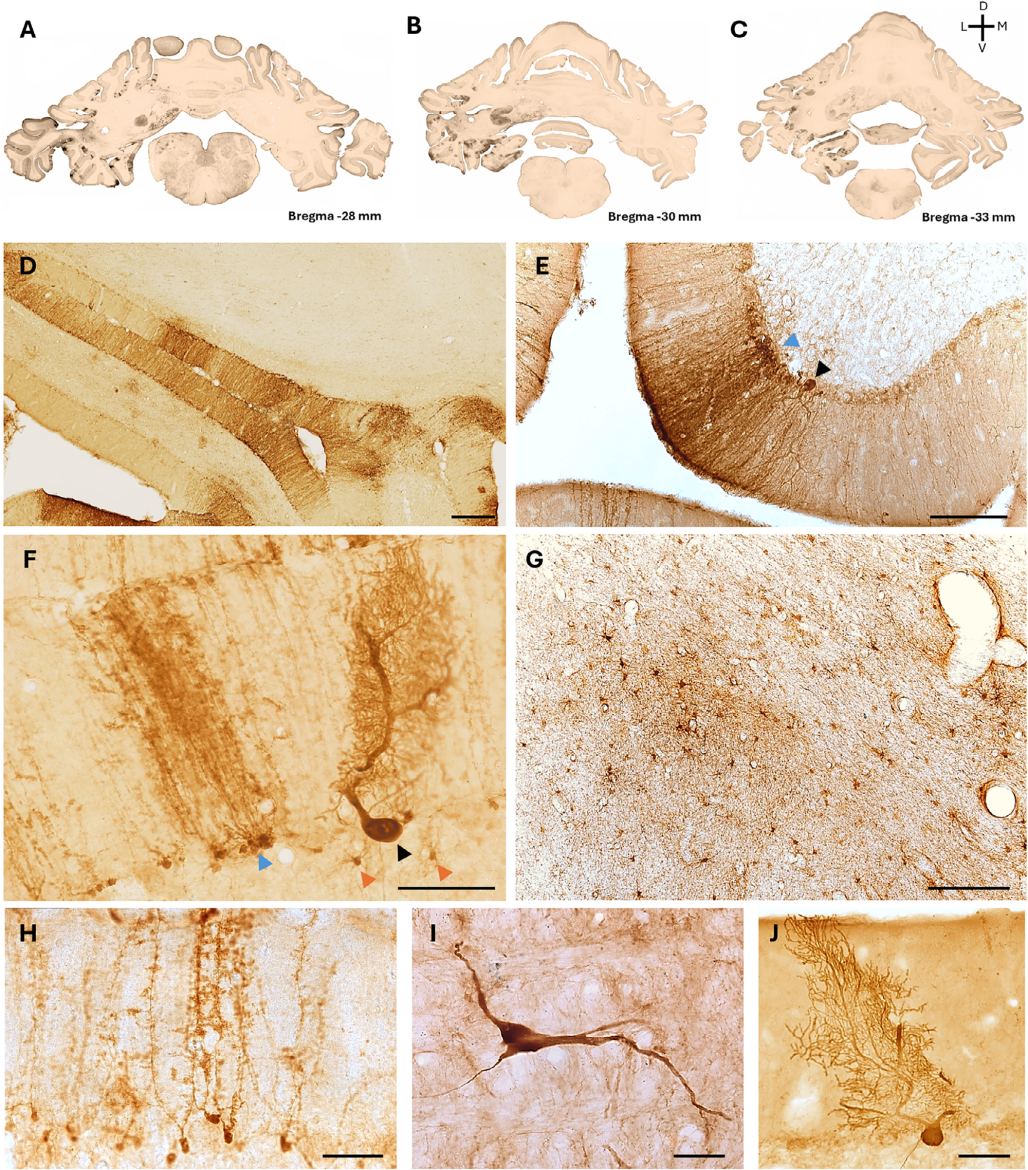
Cerebellar delivery of scAAV9‐CBA‐GFP in M2. (A–C) Pseudocolor, low‐magnification coronal sections through the rostrocaudal axis show the distribution and relative density of GFP immunoreactivity. The bregma level of each section, based on the stereotaxic atlas of Paxinos et al. [13] is indicated in the lower right corner of each panel. (D) Mid‐magnification views show a widespread GFP expression pattern across cortical regions of the BBB‐opened cerebellar hemisphere. (E,F) Representative images illustrate GFP immunostaining in Purkinje cells (black arrows), Bergmann glia (blue arrows), and astrocytes in the granular layer (orange arrows). (F) Note how Bergmann glial cells can be identified due to their unique morphology and location within the Purkinje cell layer. (G) High‐magnification images show GFP+ astrocytes in the white matter. (H) High‐magnification images show the Bergmann glia ascending processes that extend from the somata in the Purkinje cell layer to the pial surface through the molecular layer, (I) interneurons, and (J) Purkinje cells in the Purkinje layer. D: dorsal; V: ventral; M: medial; L: lateral. Scale bars: (D) 400 µm, (E–G) 200 µm, (H–J) 50 µm.

**FIGURE 6:**
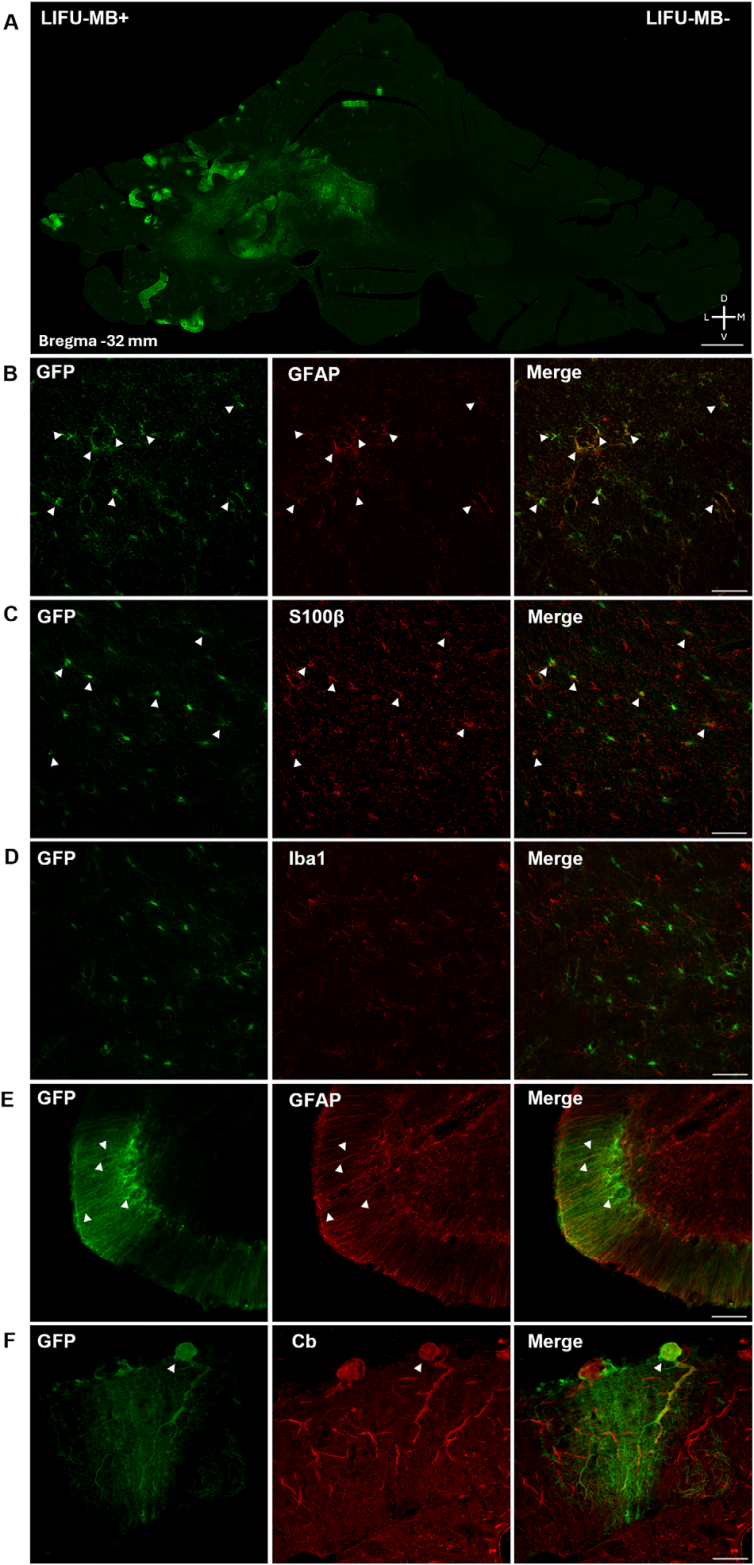
Phenotypic characterization of transduced cells in monkeys injected with scAAV9‐CBA‐GFP. (A) Panoramic view of GFP expression in a coronal cerebellar section of monkey M1, showing the treated hemisphere (LIFU‐MB+) on the left and the contralateral hemisphere (LIFU‐MB‐) on the right. Extensive GFP expression is observed only in the treated hemisphere, whereas no GFP signal is detected contralaterally. The bregma level of this section, based on the stereotaxic atlas of Paxinos et al. [13] is indicated in the lower left corner. High‐magnification images show double immunolabeling for: (B) GFAP/GFP in white‐matter astrocytes; (C) S100β /GFP in white‐matter astrocytes; (D) Iba1/GFP, showing no colocalization with GFP; (E) GFAP/GFP, showing partial colocalization in a subset of Bergmann glial fibers within the molecular layer; (F) and Cb/GFP (calbindin), demonstrating colocalization in Purkinje neurons. White arrows depict transduced cells. D: dorsal; V: ventral; M: medial; L: lateral. Scale bars: 1 mm (panoramic image), 50 µm (B–F).

Morphological characterization of GFP+ cells in the two monkeys injected with scAAV9‐CBA‐GFP (M1 and M2) revealed abundant GFP+ glial cells throughout the white matter and within the granular and molecular layers of the cerebellar cortex, including Bergmann glia (Figures 4 and 5; the original section corresponding to the pseudocolor Figure 4B is shown unaltered in Figure 6A with immunofluorescent staining for comparison). Astrocytes in the granular layer were distinguished by their numerous fine, highly branched processes. Bergmann glial cells were identified by their unique shape and location within the Purkinje cell layer and by their characteristic ascending processes extending to the pial surface (Figures 4 and 5). GFP+ neurons, including Purkinje cells, were also identified, with widespread labeled processes extending across the entire opened hemisphere of the cerebellum (Figures 4 and 5). In contrast, no measurable GFP expression was detected in the contralateral hemisphere (Figures 4, 5, and 6A; Figure S4). GFP quantification revealed up to a 3‐ and 2‐fold increase in GFP expression in the opened hemisphere compared with the non‐opened hemisphere in monkeys M1 and M2, respectively (Figure S5A). No meaningful GFP staining was observed in other brain regions or in the spinal cord (Figure S5). Only occasional, faintly stained isolated cells, mainly astroglial, were detected (Figures S4 and S5). In monkey M2, in which the opening extended into the underlying brainstem (see Figure 1), GFP+ cells were also observed in the brainstem, exclusively in the opened hemisphere (Figure S5G,J).

Morphological characterization of GFP+ cells confirmed the phenotype of the transduced cells. Although no universal marker exists for astroglia [14], double immunostaining showed that glial cells generally colocalized with the astroglial markers GFAP (Figure 6B) and S100 calcium‐binding protein B (S100β) (Figure 6C), but not with the microglial marker Iba1 (Figure 6D). Bergmann glia cells only partially colocalized with GFAP (Figure 6E). Double immunostaining for calbindin confirmed its colocalization in Purkinje cells, as previously reported [15] (Figure 6F).

**FIGURE 7:**
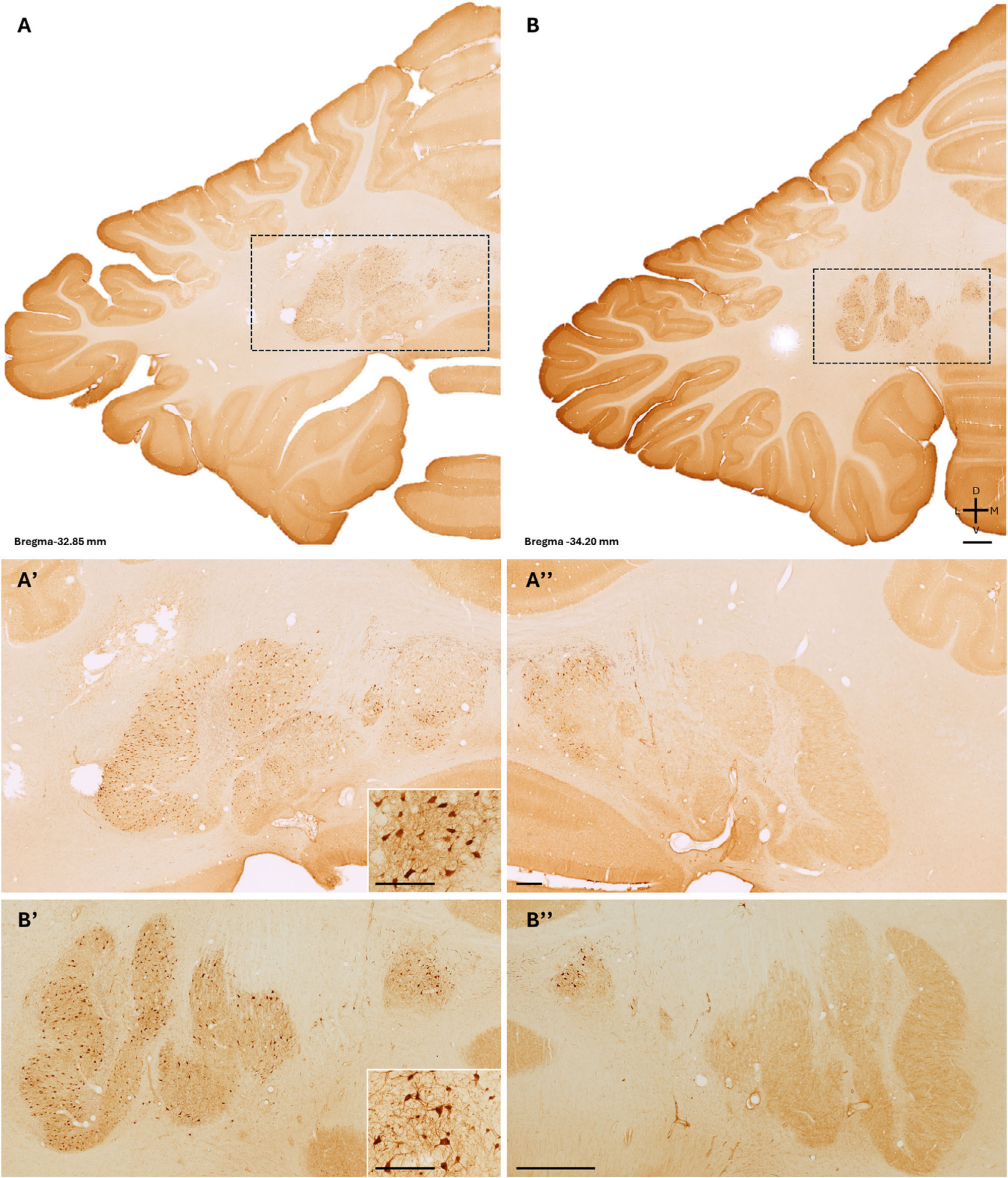
Cerebellar delivery of ssAAV9‐CMV‐mCherry in M3. (A,B) Low‐magnification images show *mCherry* immunostaining in the treated hemisphere of the cerebellum of monkey M3. The bregma level of each section, based on the stereotaxic atlas of Paxinos et al., [13] is indicated in the lower left corner. *mCherry* expression is absent in the cerebellar cortex but prominently detected in neurons of the deep cerebellar nuclei. Dashed boxes indicate regions corresponding to the magnified images shown below (A′, A″ and B′, B″). Medium‐ and high‐magnification views of *mCherry*‐labeled cells are shown in the treated (A′, B′) and contralateral (A″, B″) cerebellar hemispheres. Insets show enlarged views of representative labeled neurons. D: dorsal; V: ventral; M: medial; L: lateral. Scale bar: 1 mm.

**FIGURE 8:**
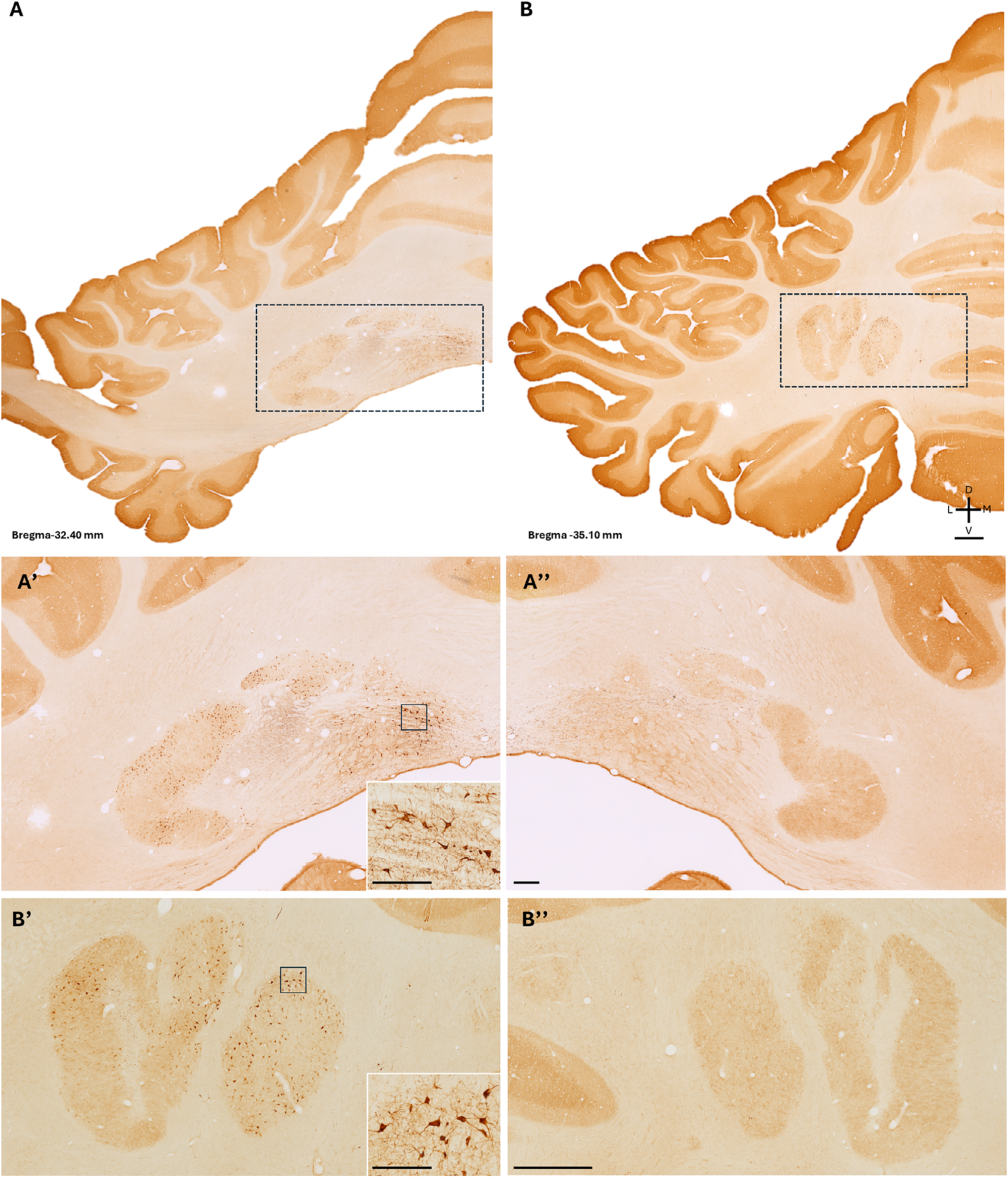
Cerebellar delivery of ssAAV9‐CMV‐mCherry in M4. (A,B) Low‐magnification images show *mCherry* immunostaining in the treated hemisphere of the cerebellum of monkey M4. The bregma level of each section, based on the stereotaxic atlas of Paxinos et al., [13] is indicated in the lower left corner. *mCherry* expression is absent in the cerebellar cortex but prominently detected in neurons of the deep cerebellar nuclei. Dashed boxes indicate regions corresponding to the magnified images shown below (A′, A″ and B′, B″). Medium‐ and high‐magnification views of *mCherry*‐labeled cells are shown in the treated (A′, B′) and contralateral (A″, B″) cerebellar hemispheres. Insets show enlarged views of representative labeled neurons. D: dorsal; V: ventral; M: medial; L: lateral. Scale bar: 1 mm.

**FIGURE 9:**
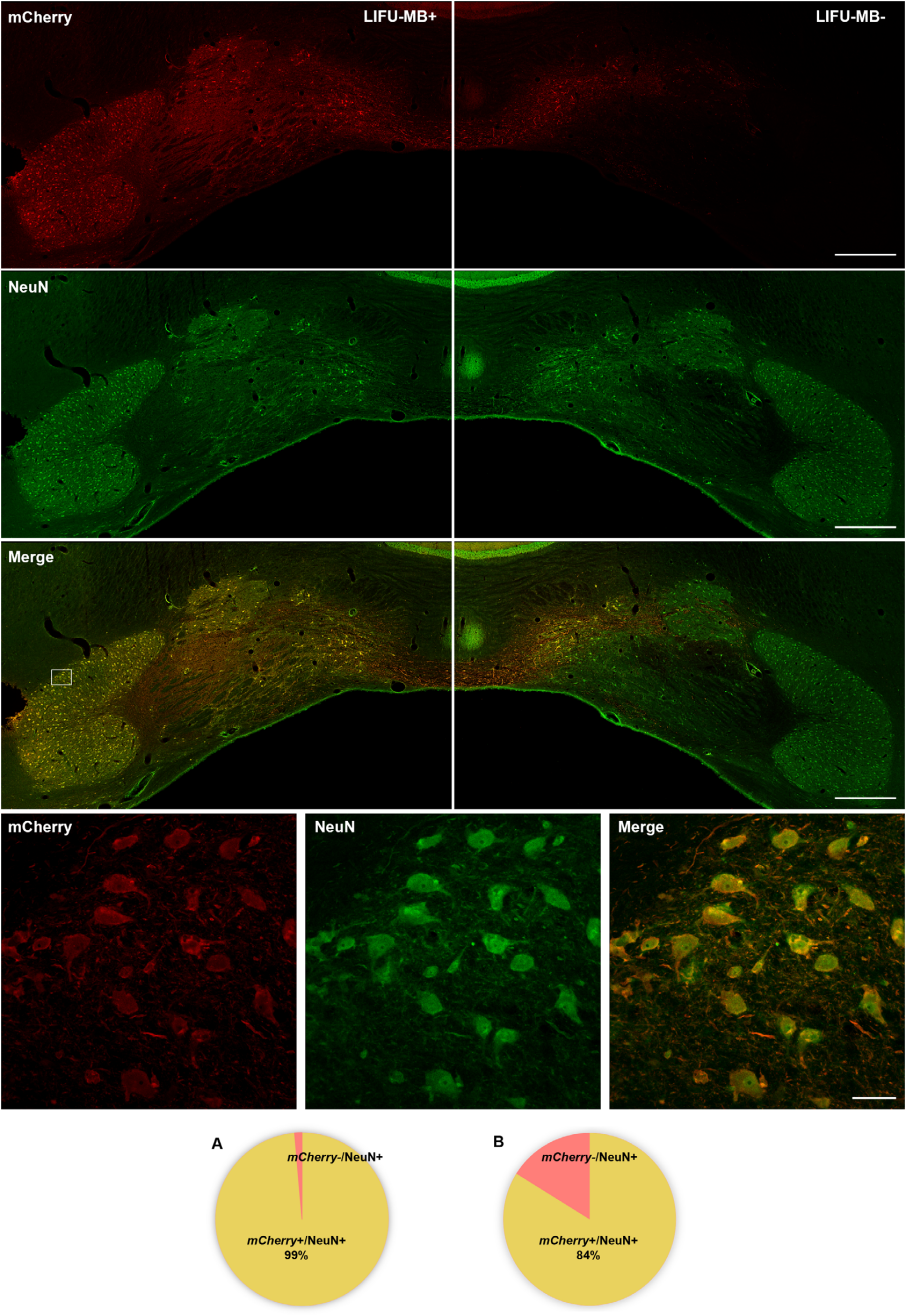
Phenotypic characterization of transduced cells in monkeys injected with ssAAV9‐CMV‐mCherry. Medium‐magnification images show a representative section of *mCherry*/NeuN immunostaining in the treated (LIFU‐MB+) and untreated (LIFU‐MB‐) cerebellar hemispheres of monkey M3. The white box indicates the region shown at higher magnification. (A,B) Pie chart showing the number of neurons transduced (*mCherry*+) of the total NeuN+ neurons in the treated hemisphere, (A) 99% in M3 and (B) 84% in M4. Scale bars: 200 µm (medium‐magnification) and 50 µm (high‐magnification).

To test the ability of ssAAV vectors, which can accommodate larger transgenes, to cross the BBB using LIFU‐MB, two monkeys (M3 and M4) were injected with ssAAV9‐CMV‐mCherry. Assessment of these two monkeys revealed abundant *mCherry+* neuronal cell bodies but no glial cells (Figures 7 and 8). *mCherry+* neuronal cell bodies were almost exclusively located in the deep cerebellar nuclei (dentate, emboliform, globose, and fastigial nuclei) of the opened cerebellar hemisphere (Figures 7 and 8). No differences in transduction were observed among the different nuclei; any apparent variation reflected the extent of BBB opening. For example, in monkey M3, where the opening extended slightly into the contralateral hemisphere, *mCherry*+ neurons were also detected in the fastigial nucleus, the nucleus closest to the midline (Figures 7 and 9). Whole‐cerebellum neuronal mapping and quantification revealed up to 2,263 *mCherry+* neurons in M3 and 1,598 *mCherry+* neurons in M4 within the targeted cerebellum, while no labeled neurons were detected in the contralateral cerebellum (Figure S5B).

Double immunostaining confirmed that all *mCherry+* cells colocalized with the neuronal marker NeuN, but not with Iba1, GFAP, or S100β (Figure 9). A few weakly‐stained Purkinje cell bodies (and not their dendrites) were occasionally scattered in the Purkinje layer of the cerebellar cortex. Quantification of double immunostaining for NeuN and *mCherry* showed that 99% (Figure 9A) and 84% (Figure 9B) of NeuN+ neurons in the deep cerebellar nuclei of sections corresponding to the opened regions in each monkey, respectively, were efficiently transduced, indicating near‐complete neuronal transduction within the opened region. No staining was observed in other brain regions or in the spinal cord, except for a few axons detected in the superior cerebellar peduncle at the level of the pyramidal decussation (Figure S5). Overall, scAAV9‐CBA‐GFP transduced substantially more cells in non‐opened regions of the brain than did ssAAV9‐CMV‐mCherry (Figures S4 and S5).

Collectively, across all four monkeys, vector expression was confined to regions that showed Gd signal enhancement, although the extent of AAV transduction did not fully match the volume of Gd enhancement (Figure S6). In monkeys injected with scAAV9‐CBA‐GFP, the GFP quantification closely corresponded to the volume of Gd enhancement. In contrast, monkeys injected with ssAAV9‐CMV‐mCherry showed similar levels of AAV transduction despite large Gd enhancement in M3 and smaller enhancement in M4. This discrepancy appears to reflect vector tropism, as transduction in M3 and M4 was restricted to the deep cerebellar nuclei, with no cortical transduction observed. Nevertheless, monkey M3 had more transduced neurons than M4 (Figure S5B).

## 3 Discussion

This study demonstrates successful, non‐invasive, and focal gene delivery of ssAAV9 and scAAV9 vectors into the cerebellum of adult macaque monkeys following intravenous injection and BBB opening with LIFU‐MB. Using the ssAAV9‐CMV‐mCherry vector, we have successfully achieved near‐complete gene transduction of all neurons within the targeted deep cerebellar nuclei, which shows that application of suitable viral vectors enables highly focal and almost complete transduction of a brain region in a non‐invasive manner. This work advances the research field of non‐invasive, focal gene delivery in non‐human primates, and builds on previous work in which, using the same technology, we successfully delivered vectors to subcortical nuclei involved in PD, including the midbrain and the striatum. Similar results have recently been obtained [16, 17], highlighting the efficiency of viral vector delivery to the monkey brain using systemic administration combined with LIFU‐MB, compared with systemic administration alone and with transduction levels typically observed in the rodent brain [18, 19].

The cerebellum is essential for motor coordination, and its degeneration is associated with a wide range of neurological manifestations (e.g., gait disturbances, ataxia and dysequilibrium, limb incoordination, speech difficulties, and eye movement abnormalities) [20]. Cerebellar neuronal loss leading to severe atrophy occurs in genetically defined spinocerebellar ataxias, multiple system atrophy, multiple sclerosis, hypothyroidism, and chronic alcoholism. It is also related to neoplasia, among other conditions. These conditions are not currently treatable, but gene therapy holds promise as a potential treatment, with several programs in development [12]. However, the cerebellum is difficult to access for delivering putative therapeutic agents due to its location in the posterior cranial fossa, near the brainstem. This makes direct intervention quite risky. Indeed, previous studies in monkeys have highlighted the special challenge of transfecting the cerebellum following systemic and intracerebroventricular vector injections in monkeys [21, 22]. Thus, our results pave the way for new possibilities for less invasive and more targeted treatments, including the potential for early intervention. In this context, a recent study has demonstrated the feasibility of LIFU‐MB for BBB opening and drug delivery into the cerebellum of pediatric patients [6].

Some key differences distinguish this current study from our previous one methodologically [7]. Here, we have deliberately implemented the institutional clinical protocol currently used for human BBB opening with this system [8, 9, 11], including adaptive acoustic power modulation in a closed‐loop fashion and a standard microbubble infusion scheme (1.5 ml microbubble solution diluted in 250 ml saline, yielding a microbubble concentration of 0.6%). Within this clinical framework, we explored only modest adjustments of target cavitation dose (0.1–0.3) and microbubble infusion rate (0.02 to 0.05ml/s), keeping sonication duration (100s) and focal spot spacing (1.5mm) comparable to our earlier work. This LIFU‐MB protocol was associated with MRI and histological alterations in several target locations, as recently reported in another study in monkeys [23]. Such hypointensities have been frequently observed in studies involving BBB opening in humans and have not been linked to any neurological manifestation or adverse effects [8, 11, 24, 25, 26, 27]. Although these abnormal signals typically evolve toward reduction or resolution [24, 26, 28], they can be largely prevented by optimizing ultrasound parameters and microbubble dosage [7, 29]. Thus, we would like to emphasize the importance of controlling factors related to ultrasound energy delivery, cavitation dose, and microbubble infusion strategies to achieve optimal results while minimizing tissue damage [30, 31].

Another fundamental difference in this study is the viral vectors and promoters used. In our previous study, we utilized AAV9‐PHP.eB and AAV9.2‐PHP.eB under the human synapsin promoter [7]. In this study, we employed two AAV9 vectors with similar genome size but different configuration and transgene: a scAAV9‐CBA‐GFP and a ssAAV9‐CMV‐mCherry. ssAAV genomes require host‐cell DNA synthesis to convert the single‐stranded viral genome into a transcriptionally competent double‐stranded form. In contrast, scAAV vectors package a genome capable of folding into a double‐stranded configuration without host‐mediated synthesis, which leads to faster onset and higher efficiency of transduction [32]. Accordingly, scAAV vectors are generally much more efficient than ssAAV vectors at mediating transgene expression in the adult brain following intravenous injection [22, 32, 33]. However, this increased efficiency comes at the cost of a reduced packaging capacity for foreign DNA [32, 34]. The scAAV9‐CBA‐GFP vector yielded substantial transduction across the entire opened region of the cerebellum, primarily targeting glial cells plus a few neurons. By contrast, the ssAAV9‐CMV‐mCherry vector resulted in exclusive neuronal transduction, largely restricted to the deep cerebellar nuclei, with minimal labeling of cerebellar cortical neurons except for a few Purkinje cells, thus indicating a potential bias toward specific neuronal populations. These differences likely reflect a combination of factors, including vector type (ss vs. sc), promoter, transgene, and dose, highlighting the importance of considering all these parameters when interpreting transduction efficiency and cellular tropism [34, 35].

The scAAV9 vector is commonly used in gene therapy and is well known for its ability to transduce not only glial cells but also neurons, particularly in monkeys [22, 33]. However, its cellular preference depends on the central nervous system region and the method of delivery [36, 37]. For example, following systemic injection with mannitol, the scAAV vectors showed widespread transgene expression in both neurons and glial cells, whereas no transgene expression was observed with the ssAAV vector [33]. Moreover, a direct comparison between scAAV‐CAG‐PHP.eB and ssAAV‐CAG‐PHP.eB (using the same promoter and transgene) following systemic administration demonstrated that the two vectors produce distinct transduction patterns depending on the brain region: the scAAV vector showed a higher proportion of expression in non‐neuronal cells, while the ssAAV vector exhibited predominantly neuronal transduction [38]. Concerning the reporter, there is no evidence that GFP or *mCherry* reporters display cell‐type–specific segregation that could account for the marked differences observed. As for promoters, even though both the CBA and the CMV promoters are considered ubiquitous, dramatic differences in cell‐specific expression patterns are observed when constructs bearing these promoters are compared in the central and peripheral nervous systems [39, 40]. In the brain and spinal cord, the CBA promoter drove strong expression in astrocytes but few neurons, whereas the CMV promoter resulted in a higher proportion of neuronal labeling [40]. Moreover, when specifically using AAV9 vectors, divergent cellular expression can occur between two similar constitutive promoters, even when all other vector components are identical [41]. Especially in the monkey brain, the CMV promoter exhibited neuron‐predominant transgene expression [42]. In marmosets, injections of AAV8 and AAV9 vectors expressing GFP under the CMV promoter into the cerebral cortex resulted in expression almost exclusively in neurons [43, 44]. Further, when administered intravenously, the AAV9 vector with the CBh promoter (a hybrid of CMV and CBA) triggered GFP expression in astrocytes of the marmoset cerebral cortex [45], but when injected directly into the brain parenchyma, the same promoter did not drive astrocytic expression [42]. Finally, blood cells and plasma proteins can also influence the in vivo pharmacokinetics of intravenously delivered vectors [46], making these interactions complex that will require careful study.

As for the widespread transduction of ssAAV9‐CMV‐mCherry in monkey M4 despite low Gd enhancement, this discrepancy could reflect a technical issue with the Gd injection or indicate that molecular and structural changes induced by LIFU‐MB in certain brain regions, such as the cerebellum, facilitate viral vector penetration. Conversely, in M3, a large Gd‐enhanced volume was observed, yet transduction remained largely restricted to the deep cerebellar nuclei, highlighting the role of vector tropism in shaping transgene expression patterns. Studies using LIFU‐MB in mice have demonstrated that intrinsic properties of the targeted brain tissue influence AAV biodistribution, diffusion, and transduction within the parenchyma [37]. A recent study in monkeys using LIFU‐MB further suggests that relative transgene expression levels and cellular tropism may vary depending on the specific brain region targeted [17]. These distinctions are important, as different diseases may require targeting specific cell populations and/or distinct anatomical regions, which may potentially limit therapeutic options. Notably, cerebellar granule neurons are widely recognized as being particularly resistant to AAV‐mediated transduction, which may further constrain effective gene delivery in this region [21, 47]. These observations also raise questions regarding the efficacy of Gd as a surrogate marker of BBB opening. A recent study in mice suggests that Gd contrast‐enhanced MRI is an imperfect predictor of AAV delivery [48]. This is consistent with our previous study, in which we observed lower levels of AAV transduction than would have been predicted based on the extent of Gd enhancement [7].

Altogether, these findings indicate that AAV vector tropism is influenced by multiple factors, including the promoter, capsid, route of administration, species, and brain region. This underscores the critical need to validate vector performance in each specific context, particularly in non‐human primates following BBB opening. It also highlights the importance of continued optimization and comparative studies with engineered AAV variants for central nervous system gene delivery [19, 37, 49, 50].

## 4 Conclusions

This study shows that LIFU‐MB enables precise, non‐invasive gene delivery to deep cerebellar regions in macaques. These results highlight both the potential of LIFU‐MB to access large, complex brain structures with focal precision and the importance of selecting optimal vector–promoter combinations. Together, they support the development of targeted gene‐based therapies for cerebellar and other neurodegenerative diseases.

## 5 Material and Methods

### 5.1 Animals

Four macaque monkeys (*Macaca fascicularis*), 3 male and 1 female, weighing 2.5 to 4 kg, aged 3 to 4 years, and sourced from Hartelust BV (Tilburg, The Netherlands), were used in this study. Animals were housed in an animal room under standard conditions and treated in accordance with the European and Spanish guidelines (86/609/EEC and 2003/65/EC European Council Directives and the Spanish Government). The experimental protocol was approved by the Ethical Committee for Research of the *Fundacion de Investigación HM Hospitales* (CEEA‐01/2022) and of *Comunidad de Madrid* (PROEX 155.1/22). Water and fresh fruit were available ad libitum. Qualified health care personnel oversaw the monkeys′ welfare throughout the studies.

### 5.2 MRI Acquisition

MRI for planning and evaluation was acquired in a 3T MR system (Discovery 750w; GE Healthcare, Milwaukee, WI). Monkeys were placed on the MR table in sphinx position, and MRI protocols were acquired using a flexible 16‐channel coil array. The baseline MRI protocol included axial T1‐weighted Fast Spoiled Gradient‐Recalled Echo (FSPGR; voxel size 0.6 × 0.6 × 0.7 mm), T2‐weighted fast spin‐echo (T2‐FSE or T2w; 0.3 × 0.3 × 1.0 mm), and susceptibility‐weighted angiography (SWAN; 0.6 × 0.6 × 0.4 mm). All images were acquired in the axial plane and aligned with the anterior commissure–posterior commissure (AC–PC) line. The follow‐up protocol used the same geometry and sequences and additionally included axial fluid‐attenuated inversion recovery (FLAIR; 0.6 × 0.6 × 2.0 mm) and diffusion‐weighted imaging (DWI; 0.7 × 0.7 × 2.0 mm). These images were used to assess tissue integrity after sonication, specifically edema (T2w and FLAIR), microhemorrhage (SWAN), and infarction (DWI). To assess the efficacy of BBB opening, a paramagnetic Gadolinium‐derived contrast agent (Gadovist, Bayer, Germany) was administered intravenously at 0.2 ml/kg, and additional post‐contrast T1‐weighted FSPGR (T1w‐Gd) images were acquired 10 min after injection. Follow‐up MRI was acquired immediately after the BBB opening procedure in all monkeys and again at 30 days after LIFU‐MB procedure. During MRI scanning, the animals were anesthetized with a mixture of ketamine (10 mg/kg) and midazolam (1 mg/kg) and maintained during the scanning period with half of the initial dose per hour.

### 5.3 LIFU‐MB Procedure

In preparation for the LIFU‐MB procedure using the clinical setup, the animal was anesthetized, the head was shaved, and an intravenous catheter was placed in the saphenous vein. Initial anesthesia was done with medetomidine (0.01 mg/kg) and a mixture of ketamine (10 mg/kg) and midazolam (1 mg/kg), and maintained during the LIFU‐MB procedure with half of the initial dose per hour. The animal was covered with a blanket and insulating material to preserve body temperature during the whole procedure.

BBB opening was performed with an MR‐guided focused ultrasound device (ExAblate Neuro; InSightec, Haifa, Israel) with a 1024‐element phased‐array transducer (220‐kHz fundamental frequency). The transducer was positioned horizontally, tilted upward, to simplify animal placement. Before loading the animal, the transducer cavity was filled with degassed, chilled water. The full procedure required approximately 120 min and included intraprocedural MRI, target definition, microbubble administration, and low‐intensity pulsed sonication. Intraprocedural MRI consisted of a 3D T1‐weighted inversion‐recovery spoiled gradient‐echo acquisition (BRAVO). The baseline MRI used for targeting was then realigned to the intraprocedural MRI using the CT as an intermediate registration step, ensuring that all three image sets were brought into the transducer reference space. All targets were placed within the left cerebellar hemisphere, distributed across two or three separate axial planes, while the right hemisphere was maintained as a control. Each plane contained between one and three targets to cover the full rostro‐caudal extent of the region. Target locations were first planned on the baseline T1‐weighted MRI and then transferred to the intraprocedural BRAVO T1‐weighted images by placing a grid of focal spots on axial slices with a fixed in‐plane spacing of 1.5 mm between spots (Figure S7). Microbubbles (Luminity, 1.5 ml in 250 ml physiological saline; final concentration 0.6%) were infused through a catheter at a constant rate of 0.05 ml/s using an MRI‐compatible infusion pump, leading to an effective microbubble infusion of 0.3 µl/s. In all cases, infusion was started 3 to 4 min before application of the first low‐intensity pulsed sonication. The closed‐loop acoustic controller was configured with a maximum acoustic power limit of 5–8 W and a target accumulated cavitation dose per spot of 0.1–0.3, estimated from microbubble‐emitted energy in the subharmonic range (110 kHz ± 40 kHz). Sonications lasted 100 s, during which the controller automatically modulated acoustic power to reach the prescribed dose at each grid spot. After every sonication, a single‐slice axial T2*‐weighted image centred on the target was acquired and visually inspected to rule out abnormalities or T2* hypointensities. If any such finding was detected, the target dose was reduced for subsequent sonications. Once the procedure was completed and all targets were sonicated, monkeys were taken off the ExAblate and transferred to the MR bed for MRI acquisition of the follow‐up protocol. All monkeys were pretreated with methylprednisolone (0.25 ml/kg, i.m.) before sonication. Based on behavior and MRI in vivo findings monkey M3 was further treated for 4 days with methylprednisolone (0.5 ml/kg, i.m.) after the treatment until full recovery.

### 5.4 Viral Vector Injection

The AAV9 vector (scAAV9) expressing green fluorescent protein (GFP) under the chicken β‐actin promoter (scAAV9‐CBA‐GFP), and the single strand AAV9 vector (ssAAV9) expressing *mCherry* under a shortened cytomegalovirus promoter (ssAAV9‐CMV‐mCherry) used in this study were produced using a transient transfection process of which the testing articles met acceptance and release criteria for in vivo research as described before [51]. The vector genome sizes (ITR‐to‐ITR) of GFP and *mCherry* closely match the wild‐type AAV genome size (scAAV9‐CBA‐GFP ∼2.1 kb and ssAAV9‐CMV‐mCherry ∼4.2 kb). Both constructs are within the packaging capacity of their respective vector types, and the encoded proteins are of similar size (∼27 kDa) [51]. Within 2 h after sonication, a vector solution was slowly infused through the saphenous vein under anesthesia: 1 × 10^1^ ^3^ vg/kg scAAV9‐CBA‐GFP in monkeys M1 and M2, and 3 × 10^1^ ^3^ vg/kg ssAAV9‐CMV‐mCherry in monkeys M3 and M4. This time frame corresponds to the post‐treatment MRI acquisition protocol to confirm successful BBB opening and discard abnormalities. The amount of AAV vectors injected was determined based on previous reports [18, 22, 51] and our own experience [7]. Detection of neutralizing antibodies was assessed in primate serum as previously described [7]. After the infusion, the monkeys were monitored until full recovery from anesthesia.

### 5.5 Postmortem Procedure

The four monkeys were anaesthetized deeply with sodium pentobarbital (10 mg/kg, i.p.) and perfused transcardially 4 weeks post‐procedure through the ascending aorta with physiological saline, followed by 4% paraformaldehyde dissolved in phosphate buffer, a series of phosphate‐buffered sucrose solutions (5 to 10 to 20%), and then cryoprotected in 30% phosphate‐buffered sucrose. Brains were blocked in the coronal plane, and spinal cords in the transverse plane, then sectioned on a freezing microtome at 40 µm to produce 10 adjacent series.

### 5.6 Nissl Staining

For Nissl staining, sections were processed as previously described [52] and incubated in 70% ethanol overnight. After a quick wash in distilled water, they were incubated at 45°C in agitation with 0.1% cresyl violet for 5 min. Following another quick wash in distilled water, the sections were incubated in sequential solutions of 70% ethanol for 1 min, 96% ethanol for 1 min, and chloroform for 10 min (in agitation) and then washed in 96% ethanol. Last, the sections were incubated in the differentiation solution under visual control and rapidly washed in 100% ethanol before being incubated in clean xylol 6 × 10 min and coverslipped with DPX.

### 5.7 Immunohistochemistry

To assess the native fluorescence of both GFP and *mCherry*, a series of sections from all animals was mounted and directly observed under a fluorescence microscope without any specific antibodies or staining. To further analyze and quantify GFP and *mCherry* immunohistochemical staining, additional sections were processed for standard immunohistochemistry. For this, free‐floating 40‐µm‐thick brain and spinal cord sections were washed in tris buffer. Inhibition of endogenous peroxidase activity was performed using a mixture of 10% methanol and 3% concentrated H_2_O_2_ for 20 min. A block solution comprising normal serum suitable for blocking non‐specific binding sites and 0.3% triton‐100X was applied for 3 h. The sections were then incubated at 4°C for a duration of 72 h with primary antibodies directed against GFP (rabbit A6455 Thermo Fisher Scientific, RRID:AB_221570; 1:1000), *mCherry* (mouse ab125096 Abcam, RRID:AB_11133266, 1:500), Iba1 (rabbit 019–19741 Wako, RRID:AB_839504, 1:100), and GFAP (mouse AB4648 Abcam, RRID:AB_449329, 1:2000). The sections were washed in tris‐buffered saline and transferred for 2 h to a solution containing the corresponding secondary biotinylated antibody (goat anti‐rabbit AP132B Chemicon, RRID: AB_11212148, 1:400) and (horse anti‐mouse BA2000 VectorLabs, AB_2313581, 1:400). Afterward, they were incubated for 45 min with the avidin‐biotin‐peroxidase complex (ABC Vectastain; Vector Laboratories). Immunohistochemical reaction products were visualized by incubating the sections with 0.05% 3,3′‐diaminobenzidine (Sigma–Aldrich) and 0.003% H_2_0_2_. The sections were then dehydrated through graded ethanols and defatted in two changes of xylene before being coverslipped with DePeX (Serva) as a mounting medium. Omission of the primary antibodies resulted in non‐immunostaining (images not shown).

### 5.8 Immunofluorescence Histochemistry

To further characterize the phenotype of the cells labeled by each viral vector, double immunofluorescence histochemistry for either GFP (rabbit A6455 Thermo Fisher Scientific, RRID:AB_221570; 1:1000 or mouse 632381 Takara, RRID:AB_2313808, 1:1000) or *mCherry* (mouse ab125096 Abcam, RRID:AB_11133266, 1:500 or goat AB004‐500 OriGene, RRID:AB_2336873, 1:500) was performed in combination with the neuronal marker NeuN (mouse MAB377 Millipore, RRID:AB_2298772, 1:500), the microglial marker Iba1 (rabbit 019–19741 Wako, RRID:AB_839504, 1:100), the astroglial markers GFAP (mouse AB4648 Abcam, RRID:AB_449329, 1:2000) and S100β (rabbit Ab52642 Abcam, RRID:AB_882426, 1:100), and calbindin, a selective marker for Purkinje cells (mouse CB300 Swant, RRID:AB_10000347, 1:7000). Sections were blocked with normal serum for 3 h before being incubated with primary antibodies for 72 h. Secondary fluorescent antibodies (Alexa Fluor goat anti‐rabbit 488, RRID: AB_143165; 1:500; Alexa Fluor goat anti‐mouse 568, RRID: AB_2534072; 1:500; Alexa Fluor rabbit anti‐mouse 488, RRID: AB_142495, 1:500; Alexa Fluor rabbit anti‐goat 568, RRID: AB_2534123, 1:500; Invitrogen) were then applied. Sections were dehydrated through graded ethanols, cleared in two changes of xylene, and coverslipped with DPX mounting medium. The immunofluorescence sections of the GFP, *mCherry*, and corresponding markers were inspected using an LSM710 confocal laser scanning microscope (Zeiss, Germany) with a magnification of ×25. In each section, 6 z‐planes were made, with a resolution of 1024×1024 ppi. For the immunohistochemistry sections, a Leica DM 2500 microscope (Leica, Germany) was used at x10 and x40 magnifications. In addition, panoramic images of the sections were taken with a Leica MICA at x10 and x20 magnification.

### 5.9 Relative Distribution and Abundance of GFP+ and mCherry Cells

To represent in a semi‐quantitative manner the presence of GFP+ neurons and neuropil elements in monkeys injected with scAAV9‐CBA‐GFP (M1 and M2), images regularly spaced at intervals of 800 µm covering the entire rostrocaudal and dorsoventral axes of the cerebellum were acquired at 10X using a MICA Leica microscope. These images were converted to pseudocolor images using the lookup tables function (Sepia) in Fiji /ImageJ (v1.53; NIH, USA). This allowed us to visualize the extension of the areas with GFP‐labeled staining. The original section corresponding to Figure 4A is shown unaltered in Figure 6A with immunofluorescent staining for comparison.

In addition, four GFP+ fluorescent sections, spaced 1200 µm apart and covering the main transduction area, were used to quantify the surface area occupied by GFP immunoreactivity via optical density (OD) analysis. All images were acquired under identical illumination and exposure settings in both monkeys (M1 and M2). Regions of interest (ROIs), including the treated and untreated hemispheres, were imaged at ×10 magnification using a Leica MICA microscope (Leica Microsystems, Germany). Background OD was measured in a 0.080 mm^2^ region of white matter within the same section of the untreated hemisphere and subtracted from each ROI measurement. A global calibration was performed using a reference image and the Uncalibrated OD function in Fiji/ImageJ (v1.53; NIH, USA). All images were processed using the same pipeline: they were converted to 8‐bit grayscale, ROIs corresponding to the treated and untreated hemispheres were delineated, and OD values were obtained as the mean gray level using the Measure function. Results were normalized to the mean of the non‐opened hemispheres of both monkeys and expressed as fold change over this mean.

In monkeys injected with ssAAV9‐CMV‐mCherry (M3 and M4) the number of cells labeled for *mCherry* were quantified using StereoInvestigator software (MBF Bioscience, Williston, VT) by mapping the whole section in a total of 20–24 sections regularly spaced at intervals of 800 µm, covering the entire rostrocaudal and dorsoventral axes of the cerebellum. First, the section and the different nuclei was outlined at 2X magnification, and then every *mCherry+* cell was marked with a digital marker under 20X magnification through the whole section.

Additionally, the percentage of neurons transduced with *mCherry* in the deep cerebellar nuclei was assessed in the opened region. To this end, the number of *mCherry+* neurons was quantified relative to the number of NeuN+ neurons in five consecutive cerebellar sections per monkey, spaced 800 µm apart, corresponding to the regions showing the greatest BBB opening, as determined by prior analysis in each animal.

To assess the expression of both viral vectors throughout the whole brain in the four monkeys, three coronal sections spanning the entire rostrocaudal axis were analyzed. Sections were spaced 4000 µm apart and included representative anterior, middle, and posterior cortical and subcortical regions.

## Author Contributions

Conceptualization: I.R., J.A.P.‐P., J.A.O., and J.B. Methodology: N.E.G., J.A.P.‐P., I.T.‐D., M.C., A.R.‐S., M.G., M.C.‐O., V.M.‐C., M.T., I.R., and J.B. Funding acquisition: J.A.P.‐P., J.A.O., and J.B. Writing – original draft: N.E.‐G., J.A.P.‐P., J.A.O., and J.B. Writing – review and editing: All authors read and approved the final version.

## Funding

This work was supported by Instituto de Salud Carlos III PFIS (FI21/000919) (N.E.‐G.), Miguel Servet (MS19/00200) and FIS (PI23/00496) (J.B.); Ministerio de Ciencia e Innovación y Universidades (PID2021‐127800OA‐I00) (J.A.P.‐P.).

## Ethics Approval

The experimental protocol was approved by the Ethical Committee for Research of the Fundacion de Investigacion HM Hospitales (CEEA‐01/2022) and of Comunidad de Madrid (PROEX 155.1/22).

## Conflicts of Interest

I.R. is an employee of Insightec Ltd. that developed and commercializes the focused ultrasound transducer used in this study. J.A.O. has been a member of the Advisory Board of Insightec Ltd. (2021–2025). The authors declare that they have no other competing interests.

## Supporting information




**Supporting File**: advs75307‐sup‐0001‐SuppMat.pdf.

## Data Availability

The data that support the findings of this study are available from the corresponding author upon reasonable request.
