# Supplementary material for: Noninvasive Focal Gene Delivery into the Cerebellum of Non‐Human Primates using Focused Ultrasound
Source: Adv Sci (Weinh). 2026 Apr 20:e75307. Online ahead of print. doi: 10.1002/advs.75307 (PMC13334610; doi:10.1002/advs.75307)
Supplement: Supplementary file 1 — Supporting File: advs75307‐sup‐0001‐SuppMat.pdf. [file ADVS-9999-e75307-s001.pdf]

ADVANCED SCIENCE Open Access

SUPPORTING INFORMATION

Noninvasive Focal Gene Delivery into the Cerebellum of Non-Human Primates using Focused Ultrasound

Noelia Esteban-García, José A. Pineda-Pardo, Inés Trigo-Damas, Marta Gutiérrez, Marta Castillo-Ortiz, Megan Carrillo, Alejandro Reinares-Sebastián, Víctor Medina-Chavarrías, Itay Rachmilevitch, Masahiko Takada, José A. Obeso* and Javier Blesa*

Correspondence: José A. Obeso (jobeso.hmcinac@hmhospitales.com); Javier Blesa (jblesa.hmcinac@hmhospitales.com)

This PDF includes:

Figure S1. Histological assessment of tissue integrity following LIFU-MB mediated BBB opening and AAV vector delivery.

Figure S2. Microglial assessment of tissue integrity following LIFU-MB mediated BBB opening and AAV vector delivery.

Figure S3. Astroglial assessment of tissue integrity following LIFU-MB mediated BBB opening and AAV vector delivery.

Figure S4. GFP and *mCherry* immunostaining in the non-opened hemispheres.

Figure S5. Expression of viral vectors in other regions of the brain and spinal cord.

Figure S6. Comparison between MRI-based blood–brain barrier (BBB) opening and histological analysis in the cerebellum following LIFU-MB.

Figure S7. Cerebellar blood-brain-barrier openings.

Table S1. Summary of sonication parameters.

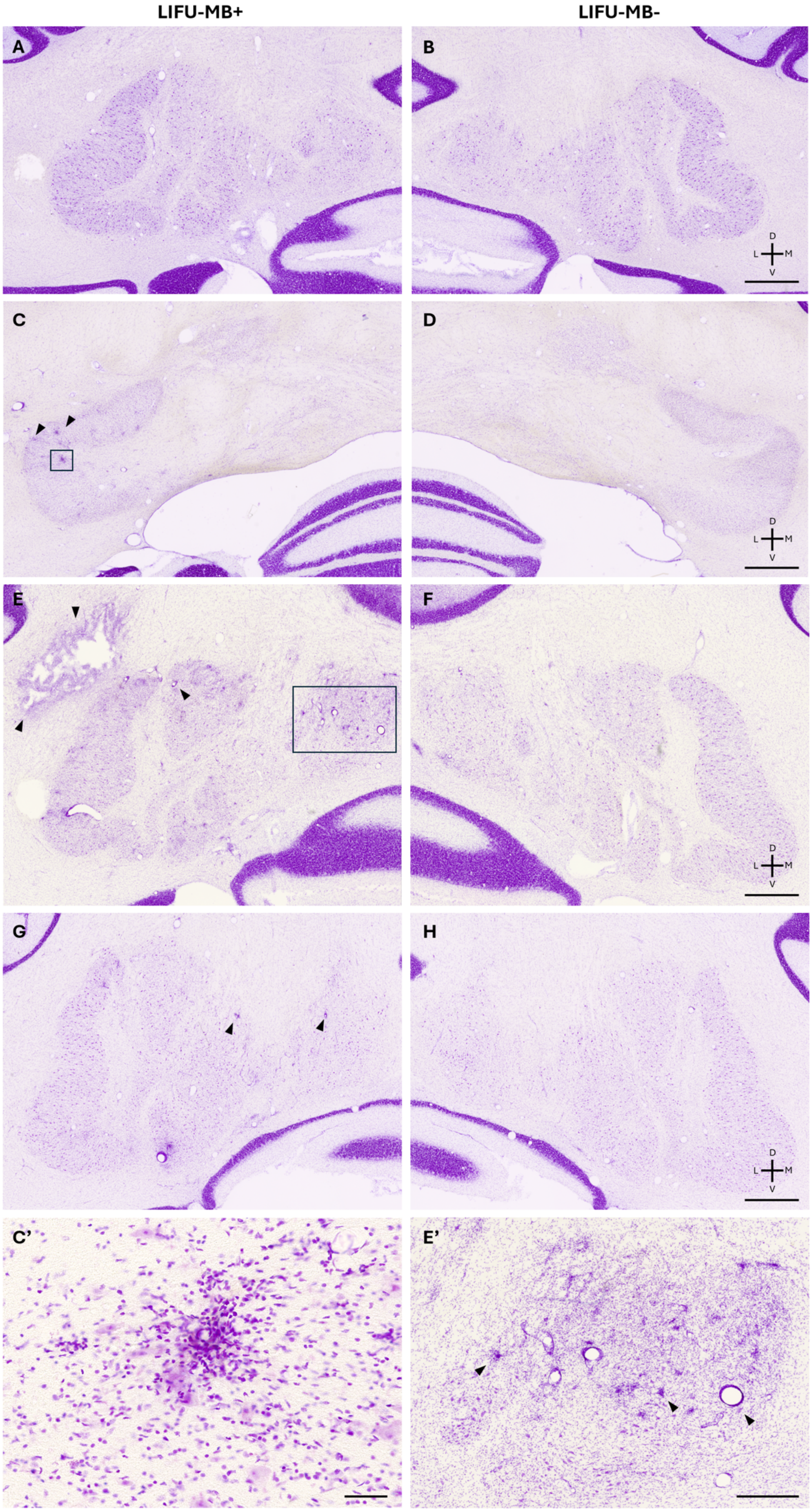

LIFU-MB+
LIFU-MB-
A
B
C
D
E
F
G
H
C'
E'
D
L
M
V

Figure S1. Histological assessment of tissue integrity following LIFU-MB mediated BBB opening and AAV vector delivery. Adjacent brain sections to those immunostained for GFP and *mCherry* were processed with Nissl staining to evaluate tissue integrity after LIFU-MB induced BBB opening and AAV vector administration. Panels show sections from monkeys M1 (A, B) and M2 (C, D), as presented in Fig. 4 and 5, and M3 (E, F) and M4 (G, H), as shown in Fig. 7 and 8. These sections are also contiguous with those stained for Iba1 (Fig. 2, Supplementary Information) and GFAP (Fig. 3, Supplementary Information). Black arrows and high-magnification views (C', E') highlight focal gliosis around blood vessels and altered vascular morphology in the cerebellum of M2, M3, and M4. D: dorsal; V: ventral; M: medial; L: lateral. Scale bars: 1 mm (A–H), 200 µm (C', E').

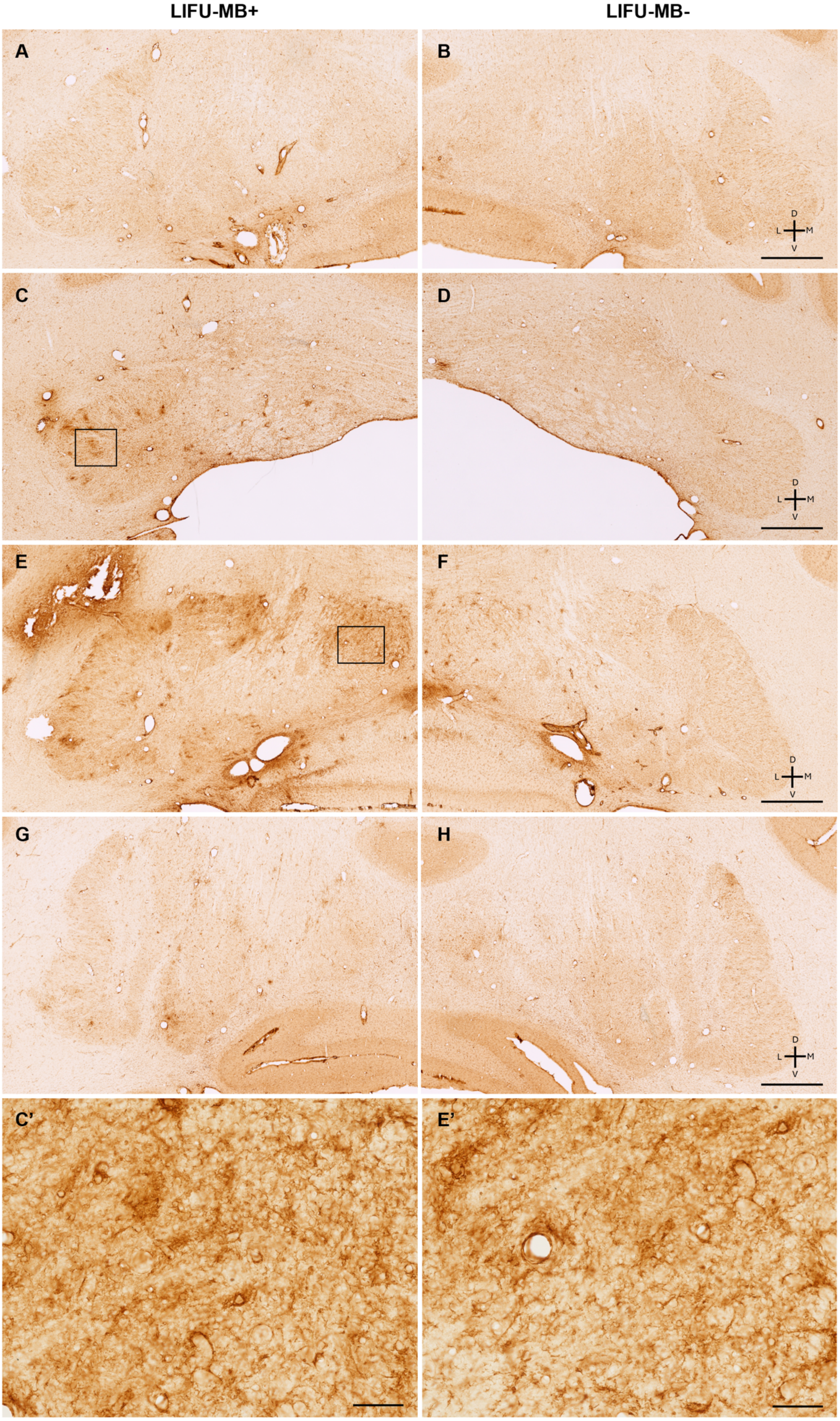

LIFU-MB+
LIFU-MB-
A
B
C
D
E
F
G
H
C'
E'
D
L
M
V

Figure S2. Microglial assessment of tissue integrity following LIFU-MB mediated BBB opening and AAV vector delivery. Adjacent brain sections to those immunostained for GFP and *mCherry* were processed with Iba1 staining to evaluate tissue integrity after LIFU-MB induced BBB opening and AAV vector administration. Panels show sections from monkeys M1 (A, B) and M2 (C, D), as presented in Fig. 4 and 5, and M3 (E, F) and M4 (G, H), as shown in Fig. 7 and 8. These sections are also contiguous with those stained for Nissl (Fig. 1, Supplementary Information) and GFAP (Fig. 3, Supplementary Information). High-magnification views (C', E') highlight increased microglial cells around blood vessels, particularly in M2 and M3 (C, E). D: dorsal; V: ventral; M: medial; L: lateral. Scale bars: 1 mm (A–H), 200 μm (C', E').

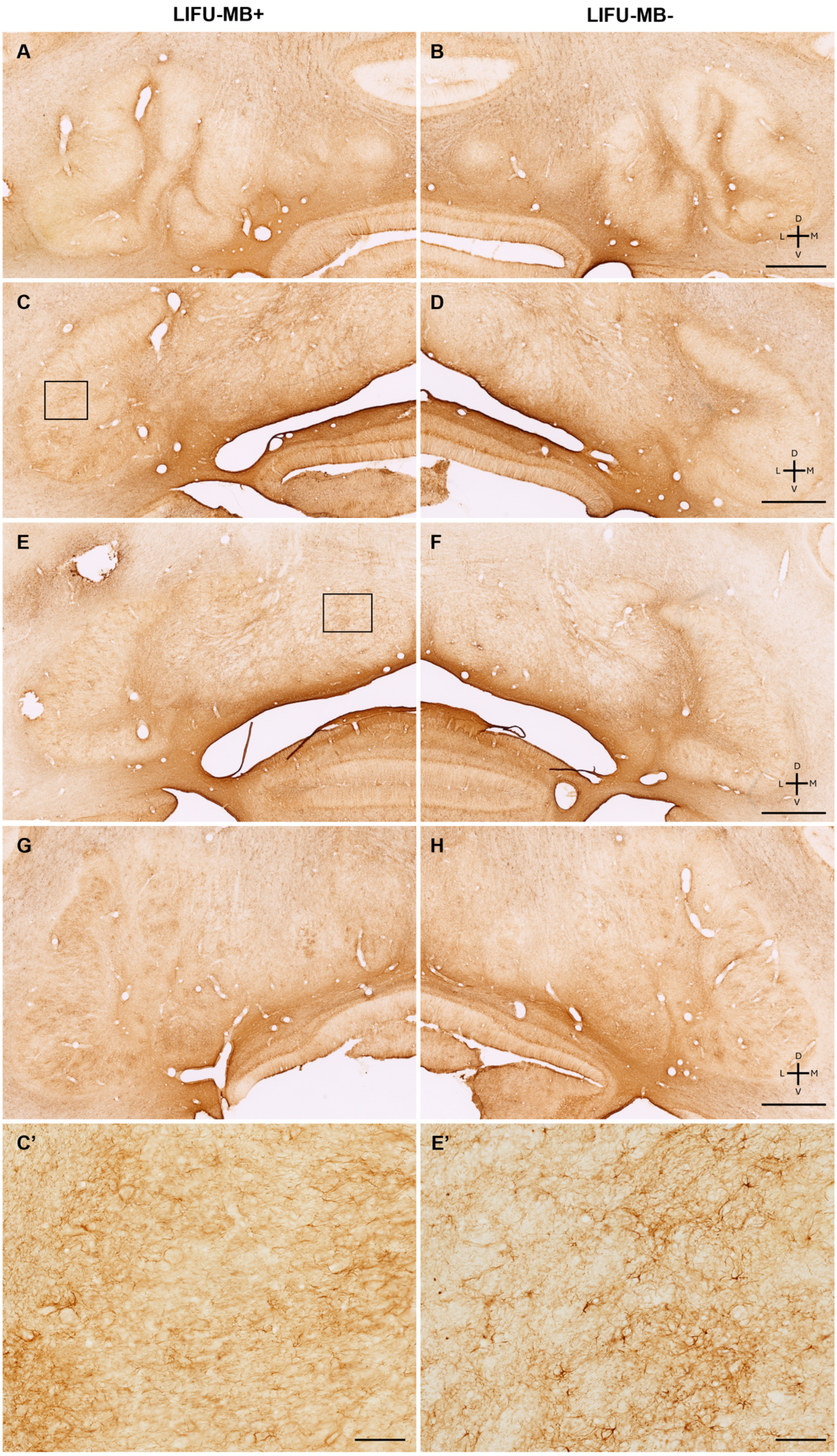
LIFU-MB+
LIFU-MB-
A
B
C
D
E
F
G
H
C'
E'
D
L
M
V

Figure S3. Astroglial assessment of tissue integrity following LIFU-MB mediated BBB opening and AAV vector delivery. Adjacent brain sections to those immunostained for GFP and *mCherry* were processed with GFAP staining to evaluate tissue integrity after LIFU-MB induced BBB opening and AAV vector administration. Panels show sections from monkeys M1 (A, B) and M2 (C, D), as shown in Fig. 2, and M3 (E, F) and M4 (G, H), as shown in Fig. 4 and 5. These sections are also contiguous with those stained for Nissl (Fig. 1, Supplementary Information) and Iba1 (Fig. 2, Supplementary Information). High-magnification views (C', E') indicate no altered astroglial expression in any monkeys, except for a focal increase of GFAP expression in M3. D: dorsal; V: ventral; M: medial; L: lateral. Scale bars: 1 mm (A–H), 200 µm (C', E').

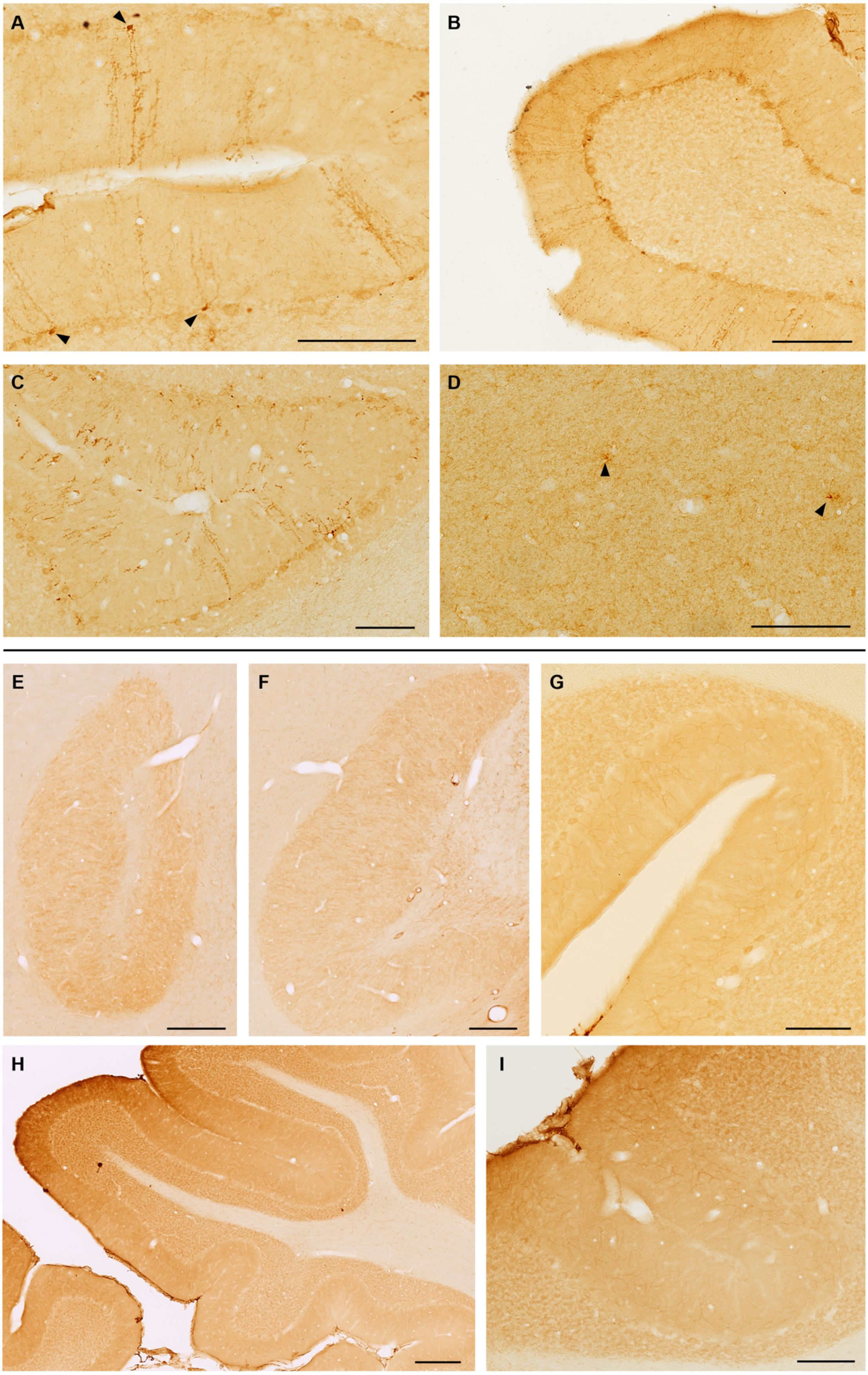
A
B
C
D
E
F
G
H
I

Figure S4. GFP and *mCherry* immunostaining in the non-opened hemispheres. Representative images of GFP immunostaining in the untreated hemisphere of the cerebellum from monkey M1 (A–D). Images correspond to the same sections shown in Figure 4. Black arrows indicate glial cells observed in the molecular layer (Bergmann glia) (A) and in the white matter (D). Notably, these cells were generally more weakly stained than those observed in the opened hemisphere. Representative images of *mCherry* immunostaining in the untreated hemisphere of the cerebellum from monkey M3 (E–I). Images correspond to the same sections shown in Figure 7. No vector expression was detected in the cerebellar cortex or deep cerebellar nuclei. Scale bars: 200 μm (A–D, G, I), 400 μm (E, F, H).

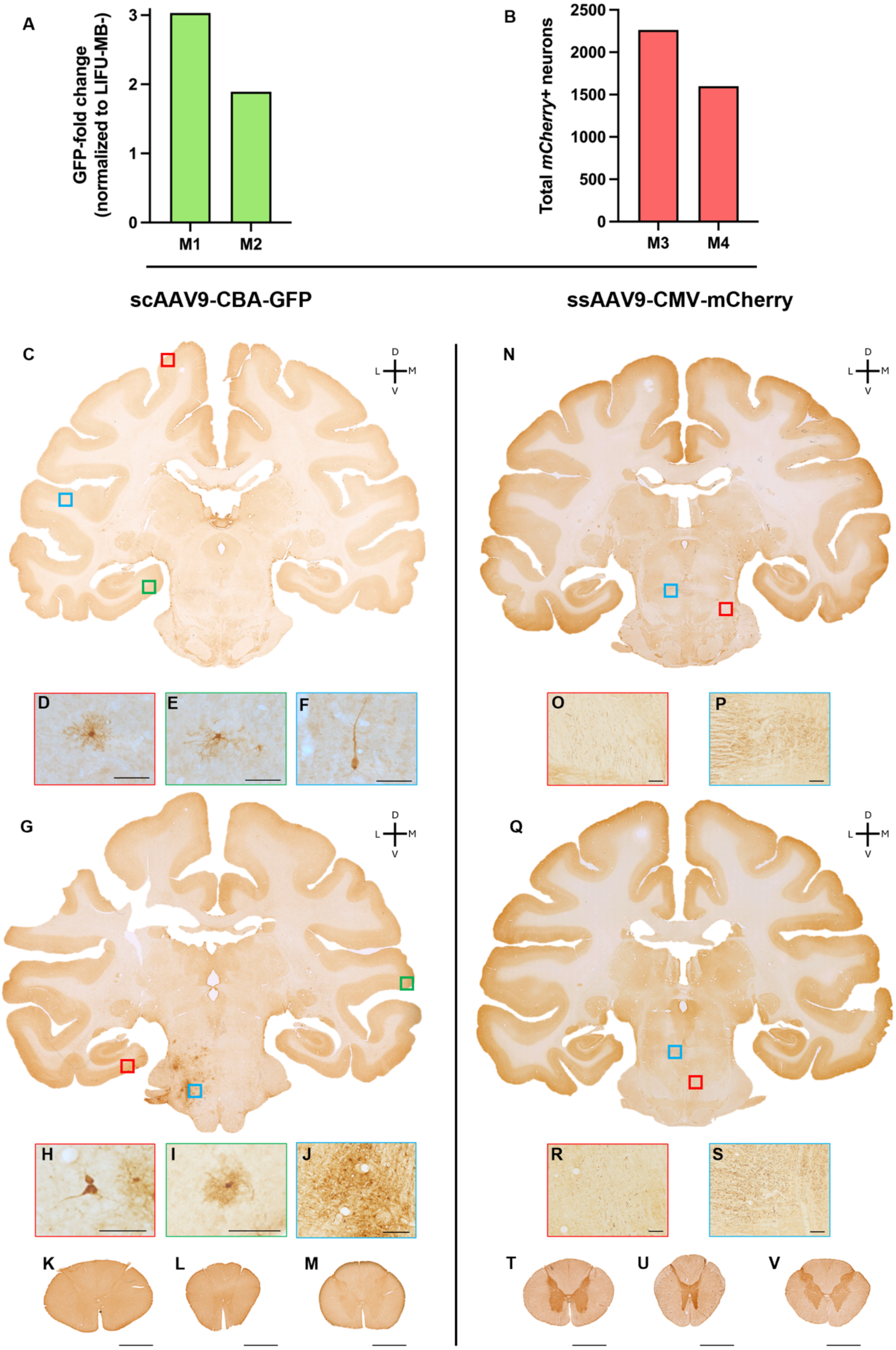
A
GFP-fold change (normalized to LIFU-MB-)
M1
M2
B
Total mCherry+ neurons
M3
M4
scAAV9-CBA-GFP
ssAAV9-CMV-mCherry
C
D
E
F
G
H
I
J
K
L
M
N
O
P
Q
R
S
T
U
V

Figure S5. Expression of viral vectors in other regions of the brain and spinal cord. Quantification of GFP expression is shown as the fold increase in optical density normalized to the untreated hemisphere (A). Quantification of the total number of *mCherry*$^+$ neurons in the deep cerebellar nuclei (B). Representative coronal sections of the brain from monkeys injected with scAAV9-CBA-GFP (C–F, monkey M1; G–J, monkey M2). Sparse GFP$^+$ astrocytes and neurons were observed in different cortical layers (D, E, F, H, I). Notably, in monkey M2, the opening extended into the underlying brainstem, and GFP$^+$ cells were detected only in the opened hemisphere (J). Representative transverse sections of the spinal cord show GFP immunolabeling in M1 at cervical (K), thoracic (L), and lumbar (M) levels, with no positive labeling observed. Representative coronal sections of the brain from monkeys injected with ssAAV9-CMV-mCherry (N–P, monkey M3; Q-S, monkey M4). No *mCherry* immunolabeling was detected, except for *mCherry*$^+$ fibers in the superior cerebellar peduncle (O, P, R, S). Representative transverse sections of the spinal cord show *mCherry* immunolabeling in M3 at cervical (T), thoracic (U), and lumbar (V) levels, with no positive labeling observed. D: dorsal; V: ventral; M: medial; L: lateral. Scale bars: 25 μm (F, G), 50 μm (B, C, D), 200 μm (H, M, N, P, Q), 1 mm (I, J, K, R, S, T).

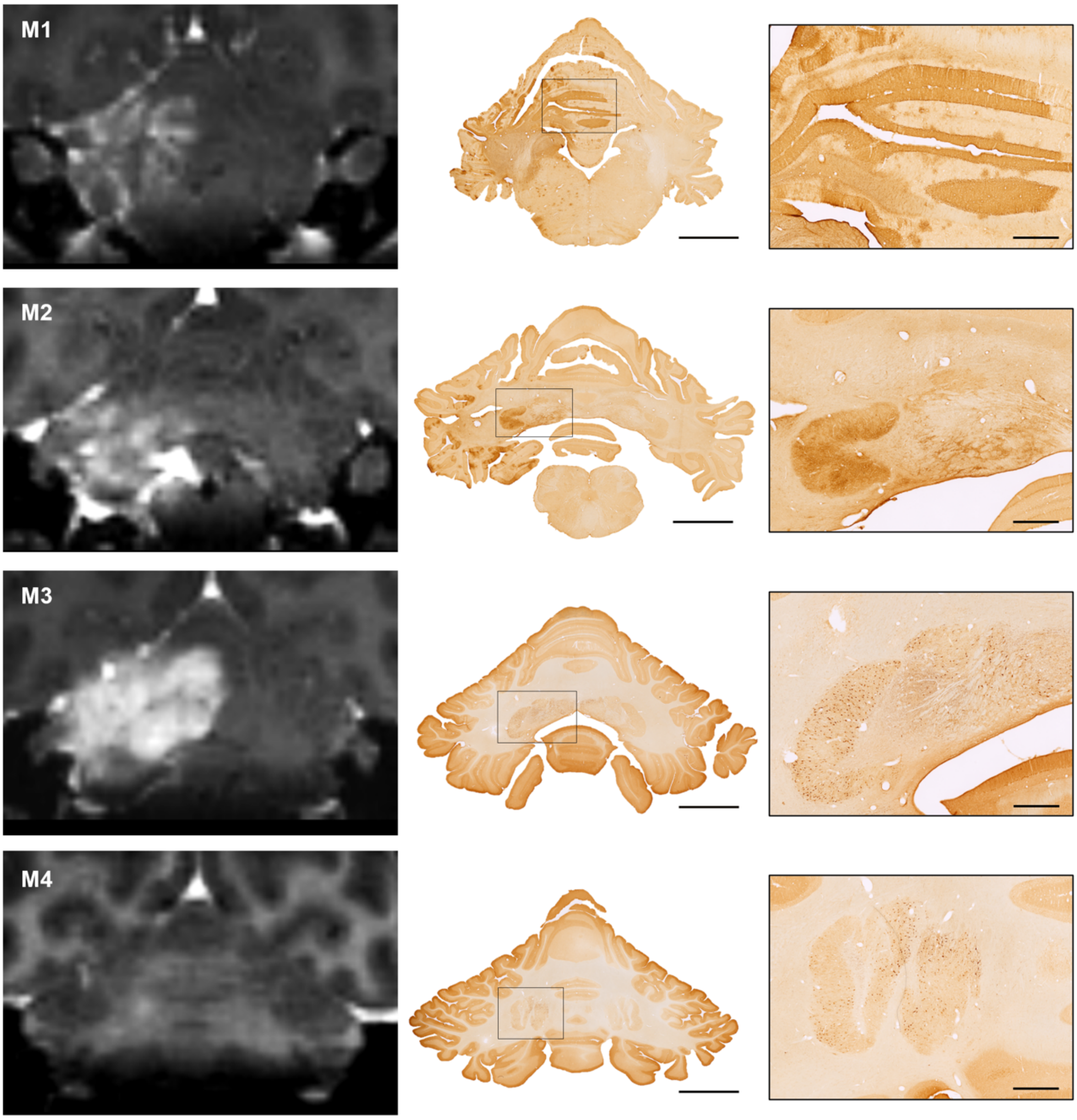

Figure S6. Comparison between MRI-based blood–brain barrier (BBB) opening and histological analysis in the cerebellum following LIFU-MB. Representative coronal T1-weighted gadolinium-enhanced (T1-w-Gd) MRI images (left column) show the extent and location of BBB opening in the four monkeys (M1–M4). Coronal histological sections stained for GFP (M1 and M2) and *mCherry* (M3 and M4), respectively (middle column), show approximately the same regions as the BBB opening and vector transduction areas. High-magnification histological images (right column) correspond to the regions indicated by squares in the middle-column images. Scale bars: 5 mm (middle column), 1 mm (right column).

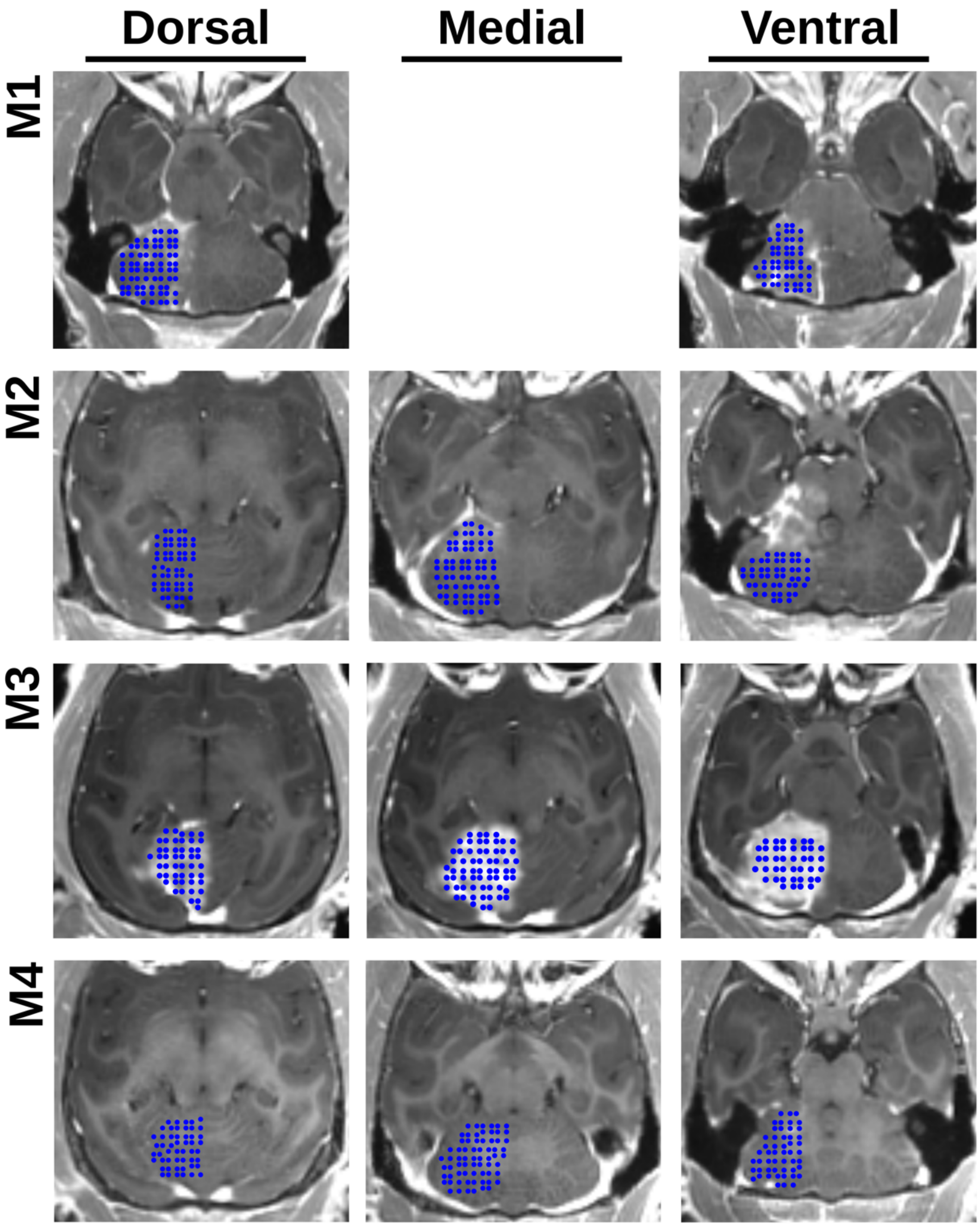

Figure S7. Cerebellar blood-brain-barrier openings. Axial contrast-enhanced T1-weighted MRI slices showing the barrier-opening targets (blue spots) used during LIFU-MB procedures in the four monkeys (M1-M4). M1 sonications were performed across two targeting planes, while sonications in all other macaques involved three targeting planes. Targeted regions correspond to areas of confirmed barrier opening, as evidenced by MRI contrast enhancement (gadolinium).

Table S1. Summary of sonication parameters for the four macaque experiments

	Targeted Region	Target	Target Description	Power (W)	Power Eff. (W)	Sonication Time (s)	Length Eff. (s)	Spots	Spacing (mm)	Target Dose	Dose
**M1**	Left Cerebellum	1	Intermediate Dorsal	8	4,8	100	100	24	1,5	0,3	0,27
		2	Posterior Dorsal	8	3,3	100	100	20	1,5	0,3	0,28
		3	Anterior Ventral	8	5	100	100	28	1,5	0,3	0,27
		4	Posterior Ventral	8	5,2	100	100	26	1,5	0,3	0,27
**M2**	Left Cerebellum	1	Dorsal Anterior	5	2,1	100	100	28	1,5	0,3	0,29
		2	Dorsal Posterior	5	3,3	100	100	31	1,5	0,3	0,29
		3	Ventral Anterior	5	2,9	100	100	31	1,5	0,3	0,28
		4	Ventral Posterior	5	4,4	100	100	17	1,5	0,3	0,24
		5	Intermediate Anterior	5	2,3	100	100	10	1,5	0,3	0,29
		6	Intermediate Med.	5	3,6	100	100	24	1,5	0,3	0,28
		7	Intermediate Posterior	5	3,8	100	100	26	1,5	0,3	0,28
**M3**	Left Cerebellum	1	Dorsal Anterior	5	2	100	100	24	1,5	0,3	0,29
		2	Dorsal Posterior	5	3,1	100	100	24	1,5	0,2	0,19
		3	Ventral Anterior	5	3,2	100	100	24	1,5	0,15	0,13
		4	Ventral Posterior	5	2,2	100	100	22	1,5	0,15	0,14
		5	Intermediate Anterior	5	1,7	100	100	19	1,5	0,15	0,14
		6	Intermediate Med.	5	2,5	100	100	26	1,5	0,15	0,14
		7	Intermediate Posterior	5	2,3	100	100	18	1,5	0,15	0,14
**M4**	Left Cerebellum	1	Dorsal Anterior	5	1,9	100	100	25	1,5	0,1	0,9
		2	Dorsal Posterior	5	3,5	100	100	26	1,5	0,1	0,8
		3	Ventral Anterior	5	3,9	100	100	27	1,5	0,15	0,13
		4	Ventral Posterior	5	3,2	100	100	24	1,5	0,1	0,08
		5	Intermediate Anterior	5	2,6	100	100	20	1,5	0,15	0,14
		6	Intermediate Med.	5	3,8	100	100	25	1,5	0,15	0,13
		7	Intermediate Posterior	5	3	100	100	22	1,5	0,15	0,13
